# Parameter optimization of vibration control system for adjacent building structures based on negative stiffness inerter damper

**DOI:** 10.1038/s41598-024-59380-1

**Published:** 2024-04-16

**Authors:** Xiaofang Kang, Jianjun Tang, Jiachen Wei, Xueqin Jiang, Ziyi Sheng, Xianzeng Shi

**Affiliations:** 1 Key Laboratory of Environmental Geotechnics, Anhui Jianzhu University, Hefei, 230601 Anhui Province China; 2 School of Civil Engineering, Anhui Jianzhu University, Hefei, 230601 Anhui Province China

**Keywords:** Adjacent building, Vibration control, Negative stiffness inerter damper, *H*_2_ norm theory, Seismic performance, Energy harvesting, Civil engineering, Energy harvesting

## Abstract

Building structures are subjected to strong earthquakes, which result in lateral collisions between them. Such collisions often cause severe structural damage and exacerbate the seismic hazard risk of building structures during earthquake events. This paper discusses the application of vibration control devices based on negative stiffness inerter damper in single-story adjacent building structures. The dynamic equations of the vibration control system containing different types of negative stiffness inerter damper under seismic excitation are established as a unified model. The H2 norm theory and Monte Carlo pattern search method are used to optimize the design parameters to improve the vibration control performance of the system, and the dynamic characteristics of the system are investigated. The results demonstrate that attaching negative stiffness inerter damper to adjacent building structures can effectively improve the overall seismic capacity reserve of the building and reduce the risk of collision of adjacent building structures; improve the robustness and stability of the system, and better reduce the displacement response of the building structure under seismic excitation. In addition, the potential of NSID-based vibration control devices to convert seismic energy into usable electricity has been investigated.

## Introduction

In recent years, as the social and economic development and the improvement of people's own living standards, the requirements for building structures are no longer limited to beautiful, but pay more attention to the safety of building structures in natural disasters. The earthquake, as a destructive natural disaster, often causes serious casualties and property damage. As a result, there has been an increased focus on the stability of building structures during earthquakes. The most common of the mechanisms for building structures to be damaged by seismic action is vibration, which occurs in building structures as seismic waves propagate through them. The vibration causes additional stresses on the structure of the building, which may result in cracked walls, leaning columns, or buckling. Past earthquake events, such as the Great Kanto Earthquake in Japan, the San Francisco Earthquake in the United States and the Wenchuan Earthquake in China, have left people with profound lessons. These disasters have prompted people to strengthen research on the seismic performance of building structures and to improve the adaptive capacity of building structures in earthquakes through scientific means, so as to reduce the damage caused by earthquakes. In the case that traditional building design and construction methods are unable to meet the high requirements of contemporary times, vibration isolation and reduction technologies have emerged to improve the seismic performance of building structures through scientific and effective design, construction methods and material selection.

It has been shown that the vibration isolation performance of a quasi-zero stiffness (QZS) system is better than the corresponding linear vibration isolation system^1,2^. The combined vibration isolation system formed by introducing a linear dynamic vibration absorber into a QZS system can effectively reduce the vibration amplitude and broaden the vibration isolation band. By tuning the frequency of the linear dynamic vibration absorber, the frequency hopping phenomenon in the QZS system can be adapted and the vibration control performance of the combined vibration isolation system can be improved^3^. The combined vibration isolation system of QZS and dynamic absorber has significantly stronger vibration reduction performance under random and impulsive excitation compared to dynamic absorber, and is suitable for ultra-low frequency vibration reduction of the primary system^4^. The addition of linear viscous damping to a conventional QZS system decreases the vibration isolation performance, and the use of dry friction element can maximize the vibration isolation performance of the system with smallest possible viscous damping^5^. The vibration isolator consisting of QZS system and shear-thinning viscous dampers solves the problem that the conventional QZS system is unable to withstand large external excitations, and thus exhibits better vibration isolation performance at medium and high frequencies^6^. A nonlinear inertance mechanism can broaden the effective isolation bandwidth of QZS and has better force transmissibility under high frequency excitation^7^. The application of inerters to both active and passive vibration isolators can effectively improve the vibration isolation performance of the system^8^. A Scotch yoke inerter implements a nonlinear inerter in a relatively simple way, which can soften the frequency response of the vibration isolator^9^. Negative stiffness damper can reduce the overall stiffness of the system, effectively reduce the influence of external excitation, so as to show better vibration isolation performance in a wider frequency range^10^. A tuned inerter damper has a larger control force in a narrower frequency range, and can effectively reduce the peak response of the system^11^. The combination of inerter and QZS system gives full play to the advantages of both devices, while mixed-connected type vibration isolators show the best vibration isolation performance^12,13^. The introduction of tuned mass negative stiffness inerter damper in base-isolated structures can significantly reduce the seismic response^14^. In practical engineering, vibration isolators need to be designed according to different engineering conditions and mechanical device requirements for vibration isolation to ensure that they can effectively reduce vibration transmission and protect equipment safety. A kind of pneumatic near-zero frequency vibration isolator composed of bellows structure, pressurized gas and incompressible liquid has the characteristics of high static and low dynamic stiffness, which can well meet the practical needs of low-frequency vibration isolation of heavy machines^15^. Compared with the metal spring isolator, the peak vibration amplitude of the new vibration isolator based on magnetorheological damper is reduced by 64%, which effectively prolongs the service life of the vibrating screen^16^. A new type of electromagnetic shunt damper simulates four different types of dampers by changing the external circuit so that the best isolation performance can be achieved in different frequency bands^17^. A 6-degree-of-freedom semi-active vibration isolation system with an additional magnetorheological damper effectively improves the linear acceleration transfer rate and vibration isolation in the resonance region^18^. Magnetorheological dampers have also been applied to semi-active controllers to reduce the transfer of helicopter rotor vibration to the fuselage and provide better vibration isolation performance in multiple directions^19^.

Inerters have the advantages of low mass and improved vibration suppression performance of the system, and thus have been the focus of research in several fields^20^. For example, towers^21–23^, milling machine^24,25^, cables^26–28^ and suspension vibration reduction systems^29,30^. The frequency of seismic waves can have an effect on the displacement response of a structure^31^. Inerters can change the intrinsic frequency of the vibration system to meet the design requirements^32^. Examples include harvesting energy from low-frequency water waves in oceans and rivers for self-powering wireless sensors^33^. Compared to the conventional electromagnetic damper, the tuned inertial mass electromagnetic damper has a significantly higher output power and better reduces the inter-story displacement of the floor^34,35^. Appropriate reduction of the auxiliary mass ratio of the damper can improve the vibration control effect of the enhanced particle inerter device^36^. The vibration control and energy harvesting performance of electromagnetic resonant shunt tuned mass damper-inerters are favored when the electromagnetic transducer is grounded^37,38^. The tuned mass damper inerter can replace the tuned mass damper to achieve better seismic isolation, and is not vulnerable to detuning effects^39–41^. In addition, the introduction of fluid inerter in TMD systems and isolators can improve the seismic performance of the system^42,43^.

Current research focuses on the vibration control of individual building structures, but the land area available for building construction is limited in metropolitan areas, and therefore the spacing of building structures is gradually decreasing to form adjacent building structures. However, collisions between adjacent building structures under seismic action present a significant hazard to building safety, so controlling the vibration response of adjacent building structures is necessary^44–46^. The inerter has a good performance in controlling adjacent buildings^47^. The presence of large relative accelerations between neighboring building structures is beneficial for inerters to generate higher resistance and thus avoid building impacts. Tuned liquid column damper inerter can mitigate the absolute acceleration of building structures under seismic excitation^48^. Inerter-based actuation schemes have excellent robustness and vibration suppression performance^49,50^. With consideration of the soil-structure interaction can reduce the desired performance of the system, such as the performance of the inerter system in reducing the displacement is weakened^51,52^. Considering background flexibility in the design formulas can consistently improve the performance of the tuned inerter damper^53^. Dampers with negative stiffness behavior have been widely investigated for structural vibration control due to their superior performance. Negative stiffness elements can reduce the apparent stiffness of the whole system, thus reducing the base shear and peak acceleration of the structure^54,55^. The combination of negative stiffness element and inerter enhances the energy dissipation capacity of the damper effectively, and different combinations of the two elements show different seismic isolation performance^56,57^. Suitable values of inertance to mass ratio and stiffness ratio can reduce the mean square response^58^. A novel smooth negative stiffness device is able to produce significant apparent weakening in the structure and does not require the additional damping to be added^59^. A coupled vibration control system with suitable negative stiffness can significantly reduce the peak transfer function of the primary structure compared to a vibration control system without negative stiffness^60^. In addition, negative stiffness can address the torsional effect generated in non-coaxial adjacent building structures, thus enhancing the nonlinear energy dissipation effects of the vibration isolation system^61^. The combination of negative stiffness elements and flexible supports realizes a larger equivalent damping force^62^. The increased deformation of the flexible connection due to negative stiffness dampers can be controlled by the rotational friction damper, and the forces transmitted to the connecting body between neighboring buildings are reduced^63^. Inerter has also been used to control the seismic response of adjacent high-rise buildings and has shown relatively good performance^48,64^.

In this paper, negative stiffness element and inerter are applied to adjacent buildings. The vibration reduction effect of two vibration control devices in adjacent building structures is revealed by comparing the dynamic characteristics of adjacent building structures with additional control devices. In the undamped case, the negative stiffness ratio and damping ratio of the vibration control structure are optimized using the *H*_2_ norm theory, in order to improve the vibration control performance of the structure. For the higher-order equations which cannot obtain analytical solutions, the Monte Carlo pattern search method is used in this paper to obtain suitable design parameters. Finally, the time domain simulation of two optimized vibration control systems for adjacent building structures have also been carried out. The main objective of this paper is to fill the research gap of negative stiffness inerter damper (NSID) applied to vibration control of adjacent building structures, and to provide a reference for subsequent research.

## Models and equations

**Figure 1:**
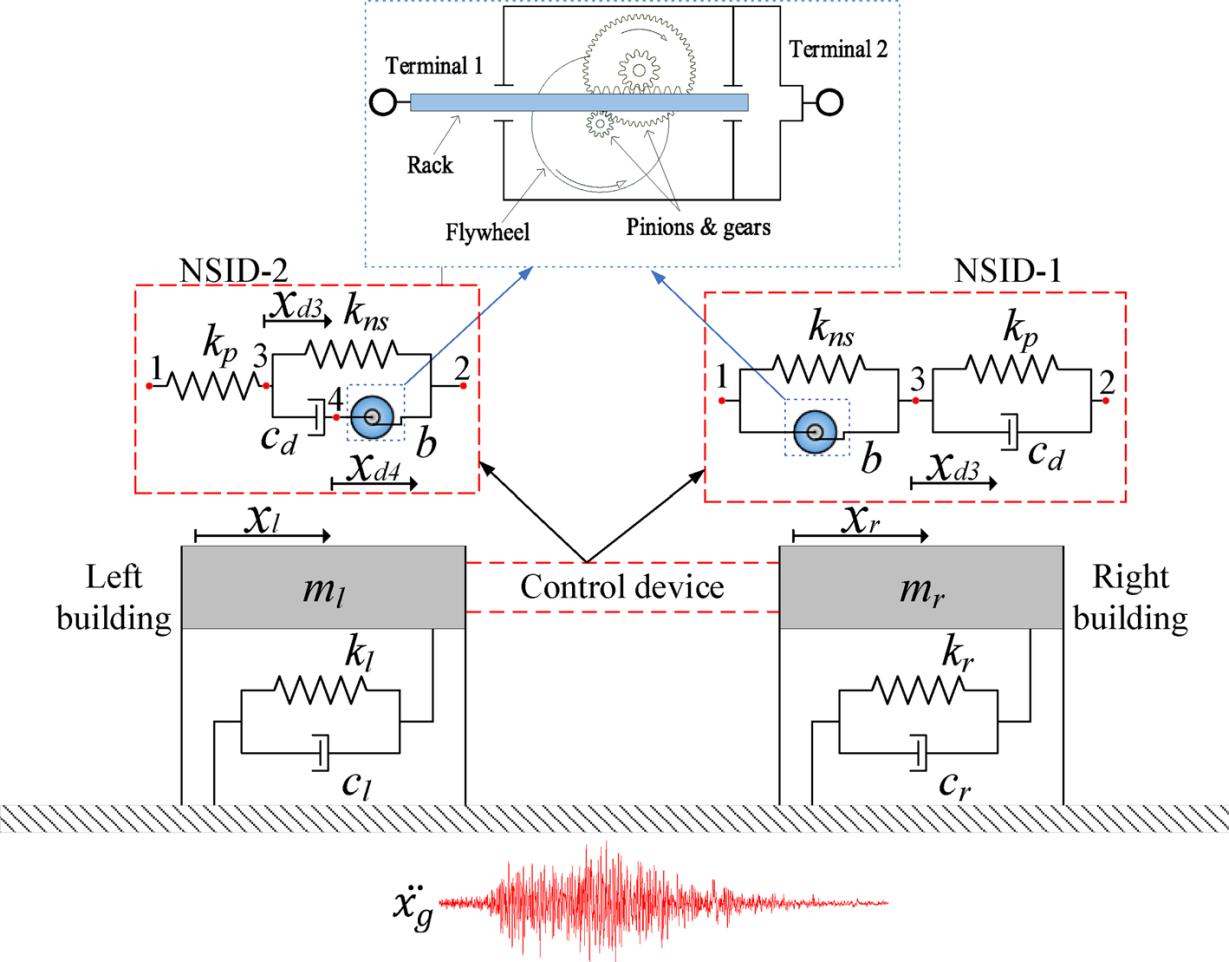
Simplified model of vibration control system for adjacent building structures based on NSID-1 and NSID-2.

A simplified model of the vibration control system for a single-story adjacent building structures researched in this paper is shown in Fig. 1. The control device connecting the two building structures consists of negative stiffness element, inerter, spring and damping element. The combination of the inerter and the negative stiffness damper is referred to as the NSID, so the two combinations in Fig. 1 are denoted as NSID-1 and NSID-2, respectively.

In the Fig. 1, ${m}_{i}$, ${c}_{i}$ and ${k}_{i}$ are the mass, damping and stiffness of the primary structure, respectively; ${x}_{i}$ is the displacement of the primary structure; $b$ is the inertance of the inerter; ${k}_{ns}$ and ${k}_{p}$ are the negative stiffness and positive stiffness of the vibration control device, respectively; ${c}_{d}$ is the damping of the vibration control device; and ${x}_{d3}$ and ${x}_{d4}$ represent the displacements at nodes 3 and 4 of the vibration control device, respectively. The subscripts $l$ and $r$ represent the left building and the right building, respectively ($i=l,r$).

An inerter is a two-terminal mechanical device which is characterized by the fact that the force at each end is proportional to the relative acceleration between the two terminals. It was demonstrated that inerter nonlinearities have a significant effect on the displacement between adjacent buildings, with friction being the main nonlinearity^65^. In this paper, a geared inerter is used as an example, where the inertance is realized by the rotation of the flywheel^66^, and the inerter forces ${{\text{F}}}_{{\text{NSID}}-1}$ and ${{\text{F}}}_{{\text{NSID}}-2}$ are calculated by the following equations:

1a $${{\text{F}}}_{{\text{NSID}}-1}=b({\ddot{x}}_{l}-{\ddot{x}}_{d3})$$

1b $${{\text{F}}}_{{\text{NSID}}-2}=b({\ddot{x}}_{r}-{\ddot{x}}_{d4})$$

The equations of motion for the two vibration control systems shown in Fig. 1 are as follows:

2 $${\text{M}}\ddot{ x}\left({\text{t}}\right)+\mathrm{C }\dot{x}\left({\text{t}}\right)+\mathrm{K }x\left({\text{t}}\right)={{\text{T}}}_{{\text{g}}} {\ddot{x}}_{g}({\text{t}})$$

where ${\text{M}}$, ${\text{C}}$, ${\text{K}}$ are the mass matrix, damping matrix, and stiffness matrix, respectively. The ${{\text{T}}}_{{\text{g}}}$ is referred to as the disturbance input vector, and ${\ddot{x}}_{g}({\text{t}})$ is the external seismic excitation acceleration. $x({\text{t}})$ is the displacement vector with respect to the ground, which is in the form shown in Eq. (3):

3 $$ {\varvec{x}}\left( {\mathbf{t}} \right)^{{{\mathbf{NSID}} - 1}} = \left[ {\begin{array}{*{20}c} {{\varvec{x}}_{{\varvec{l}}} } \\ {{\varvec{x}}_{{\varvec{r}}} } \\ {{\varvec{x}}_{{{\varvec{d}}3}} } \\ \end{array} } \right];\;{\varvec{x}}\left( {\mathbf{t}} \right)^{{{\mathbf{NSID}} - 2}} = \left[ {\begin{array}{*{20}c} {{\varvec{x}}_{{\varvec{l}}} } \\ {{\varvec{x}}_{{\varvec{r}}} } \\ {{\varvec{x}}_{{{\varvec{d}}3}} } \\ {{\varvec{x}}_{{{\varvec{d}}4}} } \\ \end{array} } \right] $$

The coefficient matrices for the two vibration control systems are shown below:

4a $$ \begin{aligned} {\mathbf{M}}^{{{\mathbf{NSID}} - 1}} = & \left[ {\begin{array}{*{20}c} {{\varvec{m}}_{{\varvec{l}}} + {\varvec{b}}} & 0 & { - {\varvec{b}}} \\ 0 & {{\varvec{m}}_{{\varvec{r}}} } & 0 \\ { - {\varvec{b}}} & 0 & {\mathbf{b}} \\ \end{array} } \right];\; \\ {\mathbf{C}}^{{{\mathbf{NSID}} - 1}} = & \left[ {\begin{array}{*{20}c} {{\varvec{c}}_{{\varvec{r}}} } & 0 & 0 \\ 0 & {{\varvec{c}}_{{\varvec{r}}} + {\varvec{c}}_{{\varvec{d}}} } & { - {\varvec{c}}_{{\varvec{d}}} } \\ 0 & { - {\varvec{c}}_{{\varvec{d}}} } & {{\varvec{c}}_{{\varvec{d}}} } \\ \end{array} } \right];\; \\ {\mathbf{K}}^{{{\mathbf{NSID}} - 1}} = & \left[ {\begin{array}{*{20}c} {{\varvec{k}}_{{\varvec{l}}} + {\varvec{k}}_{{{\varvec{ns}}}} } & 0 & { - {\varvec{k}}_{{{\varvec{ns}}}} } \\ 0 & {{\varvec{k}}_{{\varvec{r}}} + {\varvec{k}}_{{\varvec{p}}} } & { - {\varvec{k}}_{{\varvec{p}}} } \\ { - {\varvec{k}}_{{{\varvec{ns}}}} } & { - {\varvec{k}}_{{\varvec{p}}} } & {{\varvec{k}}_{{{\varvec{ns}}}} + {\varvec{k}}_{{\varvec{p}}} } \\ \end{array} } \right];\; \\ {\mathbf{R}}^{{{\mathbf{NSID}} - 1}} = & \left[ 1 \right]_{3 \times 1} \\ \end{aligned} $$

4b $$ \begin{aligned} {\mathbf{M}}^{{{\mathbf{NSID}} - 2}} = & \left[ {\begin{array}{*{20}c} {{\varvec{m}}_{{\varvec{l}}} } & 0 & 0 & 0 \\ 0 & {{\varvec{m}}_{{\varvec{r}}} + {\varvec{b}}} & 0 & { - {\varvec{b}}} \\ 0 & 0 & 0 & 0 \\ 0 & { - {\varvec{b}}} & 0 & {\varvec{b}} \\ \end{array} } \right];\; \\ {\mathbf{C}}^{{{\mathbf{NSID}} - 2}} = & \left[ {\begin{array}{*{20}c} {{\varvec{c}}_{{\varvec{l}}} } & 0 & 0 & 0 \\ 0 & {{\varvec{c}}_{{\varvec{r}}} } & 0 & 0 \\ 0 & 0 & {{\varvec{c}}_{{\varvec{d}}} } & { - {\varvec{c}}_{{\varvec{d}}} } \\ 0 & 0 & { - {\varvec{c}}_{{\varvec{d}}} } & {{\varvec{c}}_{{\varvec{d}}} } \\ \end{array} } \right];\; \\ {\mathbf{K}}^{{{\mathbf{NSID}} - 2}} = & \left[ {\begin{array}{*{20}c} {{\varvec{k}}_{{\varvec{l}}} + {\varvec{k}}_{{\varvec{p}}} } & 0 & { - {\varvec{k}}_{{\varvec{p}}} } & 0 \\ 0 & {{\varvec{k}}_{{\varvec{r}}} + {\varvec{k}}_{{{\varvec{ns}}}} } & { - {\varvec{k}}_{{{\varvec{ns}}}} } & 0 \\ { - {\varvec{k}}_{{\varvec{p}}} } & { - {\varvec{k}}_{{{\varvec{ns}}}} } & {{\varvec{k}}_{{\varvec{p}}} + {\varvec{k}}_{{{\varvec{ns}}}} } & 0 \\ 0 & 0 & 0 & 0 \\ \end{array} } \right];\; \\ {\mathbf{R}}^{{{\mathbf{NSID}} - 2}} = & \left[ 1 \right]_{4 \times 1} \\ \end{aligned} $$

The disturbance input matrix can be written as:

5 $${{\text{T}}}_{{\text{g}}}=-\mathrm{M R}$$

where ${\text{R}}$ is a column vector with all its entries equal to 1, the specific form of which is shown in Eqs. (4a and b).

## *H*_2_ optimization

The variation of parameter values in both vibration control systems can have different degrees of favorable or unfavorable effects on the final vibration control effect of the system. In order to improve the robustness and vibration control performance of the vibration control system, and thus enhance the seismic performance of the adjacent building, this paper utilizes the *H*_2_ norm theory and the Monte Carlo pattern search method to obtain the optimal parameter values of the system. To facilitate the calculation, define dimensionless parameters such as the mass ratio *μ*_*l*_ of adjacent building structures, inerter to building mass ratio *μ*_*b*_, adjacent building frequency ratio *f*_*r*_, negative stiffness ratio *α*, and so on:

6 $$ {\varvec{\mu}}_{{\varvec{l}}} = \frac{{{\varvec{m}}_{{\varvec{l}}} }}{{{\varvec{m}}_{{\varvec{r}}} }},\;{\varvec{\mu}}_{{\varvec{b}}} = \frac{{\varvec{b}}}{{{\varvec{m}}_{{\varvec{l}}} }},{\varvec{\omega}}_{{\varvec{l}}} = \sqrt {\frac{{{\varvec{k}}_{{\varvec{l}}} }}{{{\varvec{m}}_{{\varvec{l}}} }}} ,\;{\varvec{\omega}}_{{\varvec{r}}} = \sqrt {\frac{{{\varvec{k}}_{{\varvec{l}}} }}{{{\varvec{m}}_{{\varvec{l}}} }}} ,\;{\varvec{\omega}}_{{\varvec{b}}} = \sqrt {\frac{{{\varvec{k}}_{{\varvec{p}}} }}{{\varvec{b}}}} ,\;{\varvec{\xi}}_{{\varvec{l}}} = \frac{{{\varvec{c}}_{{\varvec{l}}} }}{{2 {\varvec{m}}_{{\varvec{l}}} {\varvec{\omega}}_{{\varvec{l}}} }},\;{\varvec{\xi}}_{{\varvec{r}}} = \frac{{{\varvec{c}}_{{\varvec{r}}} }}{{2 {\varvec{m}}_{{\varvec{r}}} {\varvec{\omega}}_{{\varvec{r}}} }},\;{\varvec{\xi}}_{{\varvec{b}}} = \frac{{{\varvec{c}}_{{\varvec{d}}} }}{{2 {\varvec{b}} {\varvec{\omega}}_{{\varvec{b}}} }},\;{\varvec{f}}_{{\varvec{r}}} = \frac{{{\varvec{\omega}}_{{\varvec{r}}} }}{{{\varvec{\omega}}_{{\varvec{l}}} }},\;{\varvec{f}}_{{\varvec{b}}} = \frac{{{\varvec{\omega}}_{{\varvec{b}}} }}{{{\varvec{\omega}}_{{\varvec{l}}} }},\;{\varvec{\lambda}} = \frac{{\varvec{\omega}}}{{{\varvec{\omega}}_{{\varvec{l}}} }},\;{\varvec{\alpha}} = \frac{{{\varvec{k}}_{{{\varvec{ns}}}} }}{{{\varvec{k}}_{{\varvec{p}}} }},\;{\varvec{\beta}} = \frac{{{\varvec{k}}_{{\varvec{p}}} }}{{{\varvec{k}}_{{\varvec{r}}} }},\;{\varvec{\theta}} = \frac{{{\varvec{k}}_{{\varvec{r}}} }}{{{\varvec{k}}_{{\varvec{l}}} }} $$

where, *ω* is the frequency of the ground acceleration.

### Vibration control system based on NSID-1

The solution of the dynamics equations of the vibration control system based on NSID-1 and NSID-2 can be set in the following form:

7 $$ \begin{aligned}   \ddot{x}_{i}  =  &  - X_{i} \omega ^{2} ;\dot{x}_{i}  = j\omega X_{i} ;x_{i}  = X_{i}  \\    \ddot{x}_{{d3}}  =  &  - X_{{d3}} \omega ^{2} ;\dot{x}_{{d3}}  = j\omega X_{{d3}} ;x_{{d3}}  = X_{{d3}}  \\    \ddot{x}_{{d4}}  =  &  - X_{{d4}} \omega ^{2} ;\dot{x}_{{d4}}  = j\omega X_{{d4}} ;x_{{d4}}  = X_{{d4}}  \\  \end{aligned}   $$

where, $${\varvec{i}}={\varvec{l}},{\varvec{r}}$$, $${\varvec{j}}=\sqrt{-1}$$.

In order to get the frequency characteristics of the system, the following equation can be obtained by substituting Eqs. (3, 4a, 5 and 6) into Eq. (2):

8 $$\left\{\begin{array}{c}-{X}_{l}{\lambda }^{2}-{{f}_{b}}^{2}{X}_{d3}\alpha {\mu }_{b}+{X}_{d3}{\lambda }^{2}{\mu }_{b}-{X}_{l}{\lambda }^{2}{\mu }_{b}+{X}_{l}\left(1+{{f}_{b}}^{2}\alpha {\mu }_{b}\right)+2j{X}_{l}\lambda {\xi }_{l}=-{\ddot{x}}_{g}/{{\omega }_{l}}^{2}\\ j\lambda {X}_{r}\left(2{f}_{b}{\mu }_{b}{\mu }_{l}{\xi }_{b}+2{f}_{r}{\xi }_{r}\right)-2j{f}_{b}\lambda {\mu }_{b}{\mu }_{l}{\xi }_{b}{X}_{d3}-\beta {{f}_{r}}^{2}{X}_{d3}+{X}_{r}\left(\beta {{f}_{r}}^{2}+{{f}_{r}}^{2}\right)-{\lambda }^{2}{X}_{r}=-{\ddot{x}}_{g}/{{\omega }_{l}}^{2}\\ {X}_{d3}\left(\alpha {{f}_{b}}^{2}+{{f}_{b}}^{2}\right)-{X}_{l}\alpha {{f}_{b}}^{2} -{{f}_{b}}^{2}{X}_{r}+2j{f}_{b}\lambda {\xi }_{b}{X}_{d3}-2j{f}_{b}\lambda {\xi }_{b}{X}_{r}-{\lambda }^{2}{X}_{d3}+{\lambda }^{2}{X}_{l}=0\end{array}\right.$$

Assuming that the damping ratio ${\xi }_{r}{=\xi }_{l}=0$, the displacement frequency response function of the primary structure is solved by Eq. (8):

9 $${{\text{H}}}_{l}^{{\text{NSID}}-1}=\frac{{X}_{l}(j\lambda )}{{\ddot{x}}_{g}(j\lambda )/{{\omega }_{l}}^{2}}=\frac{{{\text{b}}}_{5}^{1}{\left(j\lambda \right)}^{5}+{{\text{b}}}_{4}^{1}{\left(j\lambda \right)}^{4}+{{\text{b}}}_{3}^{1}{\left(j\lambda \right)}^{3}+{{\text{b}}}_{2}^{1}{\left(j\lambda \right)}^{2}+{{\text{b}}}_{1}^{1}{\left(j\lambda \right)}^{1}+{{\text{b}}}_{0}^{1}}{{{\text{a}}}_{6}^{1}{\left(j\lambda \right)}^{6}+{{\text{a}}}_{5}^{1}{\left(j\lambda \right)}^{5}+{{\text{a}}}_{4}^{1}{\left(j\lambda \right)}^{4}+{{\text{a}}}_{3}^{1}{\left(j\lambda \right)}^{3}+{{\text{a}}}_{2}^{1}{\left(j\lambda \right)}^{2}+{{\text{a}}}_{1}^{1}{\left(j\lambda \right)}^{1}+{{\text{a}}}_{0}^{1}}$$

10 $${{\text{H}}}_{r}^{{\text{NSID}}-1}=\frac{{X}_{r}(j\lambda )}{{\ddot{x}}_{g}(j\lambda )/{{\omega }_{l}}^{2}}=\frac{{{\text{d}}}_{5}^{1}{\left(j\lambda \right)}^{5}+{{\text{d}}}_{4}^{1}{\left(j\lambda \right)}^{4}+{{\text{d}}}_{3}^{1}{\left(j\lambda \right)}^{3}+{{\text{d}}}_{2}^{1}{\left(j\lambda \right)}^{2}+{{\text{d}}}_{1}^{1}{\left(j\lambda \right)}^{1}+{{\text{d}}}_{0}^{1}}{{{\text{c}}}_{6}^{1}{\left(j\lambda \right)}^{6}+{{\text{c}}}_{5}^{1}{\left(j\lambda \right)}^{5}+{{\text{c}}}_{4}^{1}{\left(j\lambda \right)}^{4}+{{\text{c}}}_{3}^{1}{\left(j\lambda \right)}^{3}+{{\text{c}}}_{2}^{1}{\left(j\lambda \right)}^{2}+{{\text{c}}}_{1}^{1}{\left(j\lambda \right)}^{1}+{{\text{c}}}_{0}^{1}}$$

where, the numerator (${{{\text{b}}}_{0}^{1}\sim {\text{b}}}_{5}^{1}$ and ${{\text{d}}}_{0}^{1}\sim {{\text{d}}}_{5}^{1}$) and denominator (${{\text{a}}}_{0}^{1}\sim {{\text{a}}}_{6}^{1}$ and ${{\text{c}}}_{0}^{1}\sim {{\text{c}}}_{6}^{1}$) of the displacement frequency response are detailed in Appendix A.

In this paper, the negative stiffness ratio $\alpha$ and damping ratio ${\xi }_{b}$ are defined as the design parameters of the vibration control system. The minimization of the *H*_2_ norm performance index function of the displacement frequency response function is taken as the objective so as to obtain the optimal design parameters of the system. The *H*_2_ norm of the displacement frequency response function can be defined as:

11 $$ {\text{PI}}_{i}^{{{\text{NSID}} - 1}} \left( {{\text{H}}_{i}^{{{\text{NSID}} - 1}} } \right) = \frac{{{\text{E}}\left[ {{\text{H}}_{i}^{{{\text{NSID}} - 12}} } \right]}}{{2\pi \omega _{n} {\text{S}}}} = \frac{{\left\langle {{\text{H}}_{i}^{{{\text{NSID}} - 12}} } \right\rangle }}{{2\pi \omega _{n} {\text{S}}}} $$

where, ${\text{E}}[{{{\text{H}}}_{i}^{{\text{NSID}}-1}}^{2}]$ and $\langle {{{\text{H}}}_{i}^{{\text{NSID}}-1}}^{2}\rangle$ represent the expected value and root-mean-square value of ${{{\text{H}}}_{i}^{{\text{NSID}}-1}}^{2}$ , respectively; ${\omega }_{n}$ represents the intrinsic frequency of the primary systems, represents the amplitude of the power spectral density. Among them, $\langle {{{\text{H}}}_{i}^{{\text{NSID}}-1}}^{2}\rangle ={\omega }_{n}{\text{S}}{\int }_{-\infty }^{+\infty }{\left|{{\text{H}}}_{i}^{{\text{NSID}}-1}\right|}^{2}{\text{d}}\lambda$, and the simplification of Eq. (11) can get the expression:

12 $$ {\text{PI}}_{i}^{{{\text{NSID}} - 1}}  = \int\limits_{{ - \infty }}^{{ + \infty }} {\left| {{\text{H}}_{i}^{{{\text{NSID}} - 1}} } \right|^{2} {\text{d}}\lambda }   $$

The polynomial expression for the *H*_2_ norm performance index function ${{\text{PI}}}_{i}^{{\text{NSID}}-1}$ of the displacement frequency response function ${{\text{H}}}_{i}^{{\text{NSID}}-1}$ is as follows:

13 $${{\text{PI}}}_{i}^{{\text{NSID}}-1}=\frac{{{\text{Q}}}_{i}^{{\text{NSID}}-1}}{{{\text{V}}}_{i}^{{\text{NSID}}-1}}$$

Refer to Appendix B for the calculation process of Eq. (13).

In order to obtain the optimal design parameters of the vibration control system based on NSID-1, Eq. (13) needs to satisfy the following equation:

14 $$ \frac{{\partial {\text{PI}}_{i}^{{{\text{NSID}} - 1}} }}{\partial \alpha } = \frac{{\partial {\text{PI}}_{i}^{{{\text{NSID}} - 1}} }}{{\partial \xi_{b} }} = 0 $$

However, Eq. (14) is a system of binary higher-order equations, which cannot be solved to obtain exact solutions for the design parameters. Therefore, the Monte Carlo pattern search method described in subsection "Optimization of PI using Monte Carlo pattern search method" is used in this paper to obtain suitable values of the design parameters.

### Vibration control system based on NSID-2

Substituting Eqs. (3, 4b, 5 and 6) into Eq. (2) to obtain the following equation:

15 $$\left\{\begin{array}{c}-{X}_{l}{\lambda }^{2}-{{f}_{b}}^{2}{X}_{d3}{\mu }_{b}+{X}_{l}(1+{{f}_{b}}^{2}{\mu }_{b})+2j{X}_{l}\lambda {\xi }_{l}=-{\ddot{x}}_{g}/{{\omega }_{l}}^{2}\\ -{X}_{r}{\lambda }^{2}-{{f}_{b}}^{2}{X}_{d3}\alpha {\mu }_{b}{\mu }_{l}+{X}_{d4}{\lambda }^{2}{\mu }_{b}{\mu }_{l}-{X}_{r}{\lambda }^{2}{\mu }_{b}{\mu }_{l}+{X}_{r}({{f}_{r}}^{2}+{{f}_{b}}^{2}\alpha {\mu }_{b}{\mu }_{l})+2j{{f}_{r}X}_{r}\lambda {\xi }_{r}=-{\ddot{x}}_{g}/{{\omega }_{l}}^{2}\\ -{{f}_{b}}^{2}{X}_{l}-{{f}_{b}}^{2}{X}_{r}\alpha +{X}_{d3}\left({{f}_{b}}^{2}+{{f}_{b}}^{2}\alpha \right)+2j{f}_{b}{X}_{d3}\lambda {\xi }_{b}-2j{f}_{b}{X}_{d4}\lambda {\xi }_{b}=0\\ -{X}_{d4}{\lambda }^{2}{\mu }_{b}{\mu }_{l}+{X}_{r}{\lambda }^{2}{\mu }_{b}{\mu }_{l}-2j{f}_{b}{X}_{d3}\lambda {\mu }_{b}{\mu }_{l}{\xi }_{b}+2j{f}_{b}{X}_{d4}\lambda {\mu }_{b}{\mu }_{l}{\xi }_{b}=0\end{array}\right.$$

Assuming that the damping ratio ${\xi }_{r}{=\xi }_{l}=0$, the displacement frequency response of the primary structure is solved by Eq. (15):

16 $${{\text{H}}}_{l}^{{\text{NSID}}-2}=\frac{{X}_{l}(j\lambda )}{{\ddot{x}}_{g}(j\lambda )/{{\omega }_{l}}^{2}}=\frac{{{\text{b}}}_{5}^{2}{\left(j\lambda \right)}^{5}+{{\text{b}}}_{4}^{2}{\left(j\lambda \right)}^{4}+{{\text{b}}}_{3}^{2}{\left(j\lambda \right)}^{3}+{{\text{b}}}_{2}^{2}{\left(j\lambda \right)}^{2}+{{\text{b}}}_{1}^{2}{\left(j\lambda \right)}^{1}+{{\text{b}}}_{0}^{2}}{{{\text{a}}}_{6}^{2}{\left(j\lambda \right)}^{6}+{{\text{a}}}_{5}^{2}{\left(j\lambda \right)}^{5}+{{\text{a}}}_{4}^{2}{\left(j\lambda \right)}^{4}+{{\text{a}}}_{3}^{2}{\left(j\lambda \right)}^{3}+{{\text{a}}}_{2}^{2}{\left(j\lambda \right)}^{2}+{{\text{a}}}_{1}^{2}{\left(j\lambda \right)}^{1}+{{\text{a}}}_{0}^{2}}$$

17 $${{\text{H}}}_{r}^{{\text{NSID}}-2}=\frac{{X}_{r}(j\lambda )}{{\ddot{x}}_{g}(j\lambda )/{{\omega }_{r}}^{2}}=\frac{{{\text{d}}}_{5}^{2}{\left(j\lambda \right)}^{5}+{{\text{d}}}_{4}^{2}{\left(j\lambda \right)}^{4}+{{\text{d}}}_{3}^{2}{\left(j\lambda \right)}^{3}+{{\text{d}}}_{2}^{2}{\left(j\lambda \right)}^{2}+{{\text{d}}}_{1}^{2}{\left(j\lambda \right)}^{1}+{{\text{d}}}_{0}^{2}}{{{\text{c}}}_{6}^{2}{\left(j\lambda \right)}^{6}+{{\text{c}}}_{5}^{2}{\left(j\lambda \right)}^{5}+{{\text{c}}}_{4}^{2}{\left(j\lambda \right)}^{4}+{{\text{c}}}_{3}^{2}{\left(j\lambda \right)}^{3}+{{\text{c}}}_{2}^{2}{\left(j\lambda \right)}^{2}+{{\text{c}}}_{1}^{2}{\left(j\lambda \right)}^{1}+{{\text{c}}}_{0}^{2}}$$

where, the numerator (${{\mathbf{b}}_{0}^{2}\sim \mathbf{b}}_{5}^{2}$ and ${\mathbf{d}}_{0}^{2}\sim {\mathbf{d}}_{5}^{2}$) and denominator (${\mathbf{a}}_{0}^{2}\sim {\mathbf{a}}_{6}^{2}$ and ${\mathbf{c}}_{0}^{2}\sim {\mathbf{c}}_{6}^{2}$) of the displacement frequency response are detailed in Appendix C**.**

18 $$ {\text{PI}}_{i}^{{{\text{NSID}} - 2}} = \mathop \int \limits_{ - \infty }^{ + \infty } \left| {{\text{H}}_{i}^{{{\text{NSID}} - 2}} } \right|^{2} {\text{d}}\lambda $$

Similar to Sect. 3.1, the polynomial expression for the *H*_2_ norm performance index function ${{\text{PI}}}_{i}^{{\text{NSID}}-2}$ of the displacement frequency response function ${{\text{H}}}_{i}^{{\text{NSID}}-2}$ is as follows:

19 $$ {\text{PI}}_{i}^{{{\text{NSID}} - 2}} = \frac{{{\text{Q}}_{i}^{{{\text{NSID}} - 2}} }}{{{\text{V}}_{i}^{{{\text{NSID}} - 2}} }} $$

In order to obtain the optimal design parameters of the vibration control system based on NSID-2, Eq. (19) needs to satisfy the following equation:

20 $$ \frac{{\partial {\text{PI}}_{i}^{{{\text{NSID}} - 2}} }}{\partial \alpha } = \frac{{\partial {\text{PI}}_{i}^{{{\text{NSID}} - 2}} }}{{\partial \xi_{b} }} = 0 $$

Similar to Eq. (14, 20) also fails to obtain the exact solutions for the design parameters. Therefore, this vibration control system based on NSID-2 also requires the Monte Carlo pattern search method to obtain suitable values of the design parameters.

### Optimization of PI using Monte Carlo pattern search method

The well-known Hooke-Jeeves pattern search method mainly consists of exploratory search and pattern move, which can be used for solving problems with objective functions that are not derivable or discontinuous^67^. However, it is difficult to achieve the global optimal solution because the pattern search method is affected by the number and value of starting points when searching for the regional optimal value. Therefore, the introduction of Monte Carlo method to generate a large number of random starting points can increase the probability of searching the optimal value in the region^68,69^. Wang et al. firstly proposed Monte Carlo-based pattern search method applied to a multiple tuned mass damper system, and effectively solved the problem of multi-parameter optimization which is difficult to derive the objective function^70,71^. The parameter optimization of NSID-1 and NSID-2 is based on the input parameters (${\xi }_{l}, {\xi }_{r}, {\mu }_{l}, {\mu }_{b}, {f}_{r}, {f}_{b}$) to determine the design parameters (${\alpha }_{opt}$ and ${{\xi }_{b}}_{opt}$) ,to make the performance index function ${{\text{PI}}}_{i}^{{\text{NSID}}-1}$ and ${{\text{PI}}}_{i}^{{\text{NSID}}-2}$ as much as possible to obtain the smaller value of the process, the specific requirements of its parameter optimisation can be expressed as follows:

21 $$ \left\{ {\begin{array}{*{20}l}    {{\text{Find}}} \hfill & {\alpha _{{opt}} \;{\text{and}}\;\xi _{{b\;opt}} } \hfill  \\    {{\text{To}}\;{\text{minimize}}} \hfill & {{\text{PI}}_{i}^{{{\text{NSID}} - 1/2}} (\xi _{l} ,\xi _{r} ,\mu _{l} ,\mu _{b} ,f_{r} ,f_{b} )} \hfill  \\    {{\text{Subjected}}\;{\text{to}}} \hfill & { - 1 < \alpha  < 0} \hfill  \\    {{\text{Subjected}}\;{\text{to}}} \hfill & {\alpha  >  - \left( {1 + \theta } \right)/(1 + \theta  + \beta \theta )} \hfill  \\   \end{array} } \right. $$

where, the superscript 1/2 indicates a vibration control system based on NSID-1 or NSID-2.

**Figure 2:**
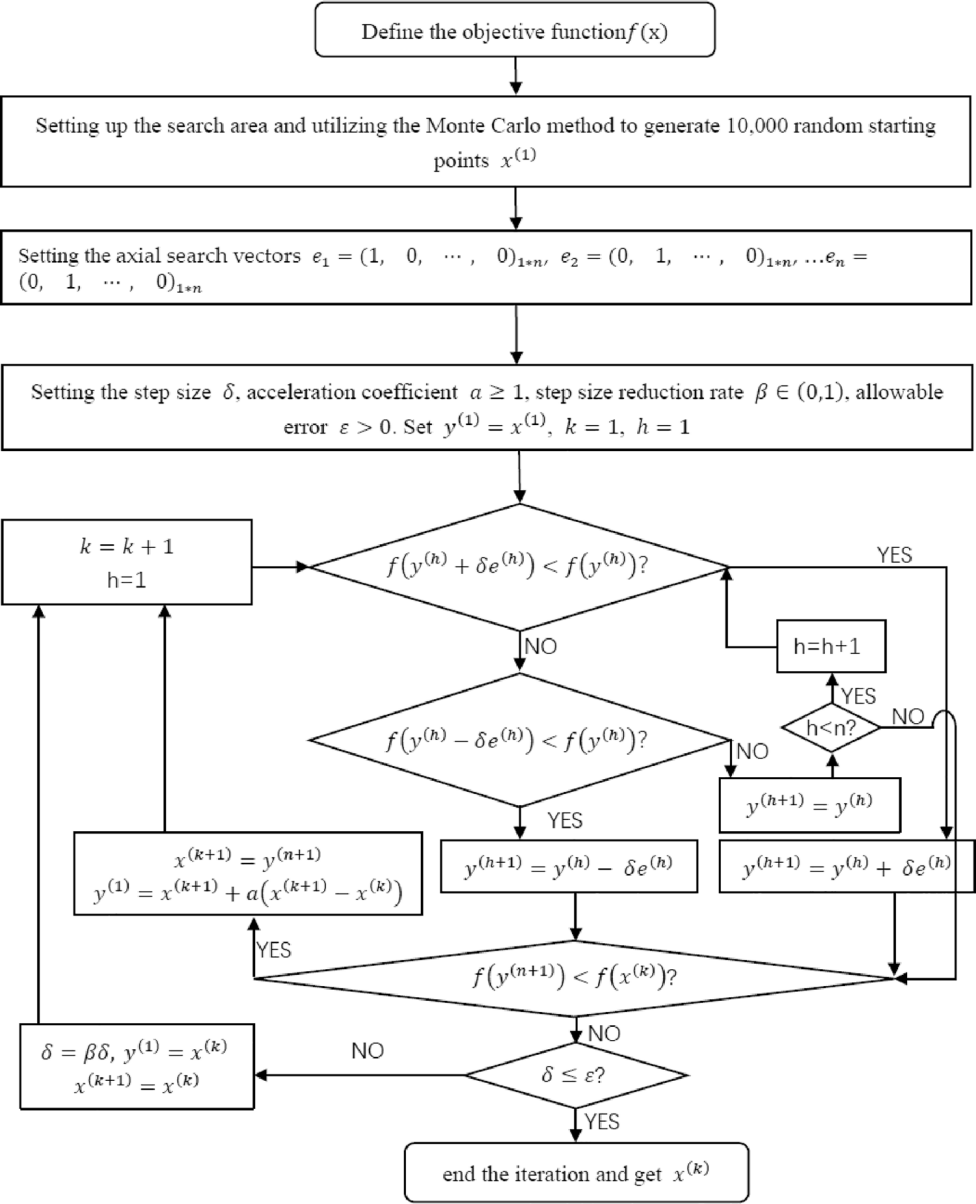
Flowchart of optimizing PI by Monte Carlo pattern search method.

After determining the frequency response function formula, parameter optimization of the negative stiffness ratio $\alpha$ and damping ${\xi }_{b}$ is required. Since the system involves more parameters, the optimal solution is mainly searched by controlling the parameter variables, which is realized by MATLAB (R2021a 9. 10. 0. 1,602,886), and the specific implementation flowchart is shown in Fig. 2.

**Figure 3:**
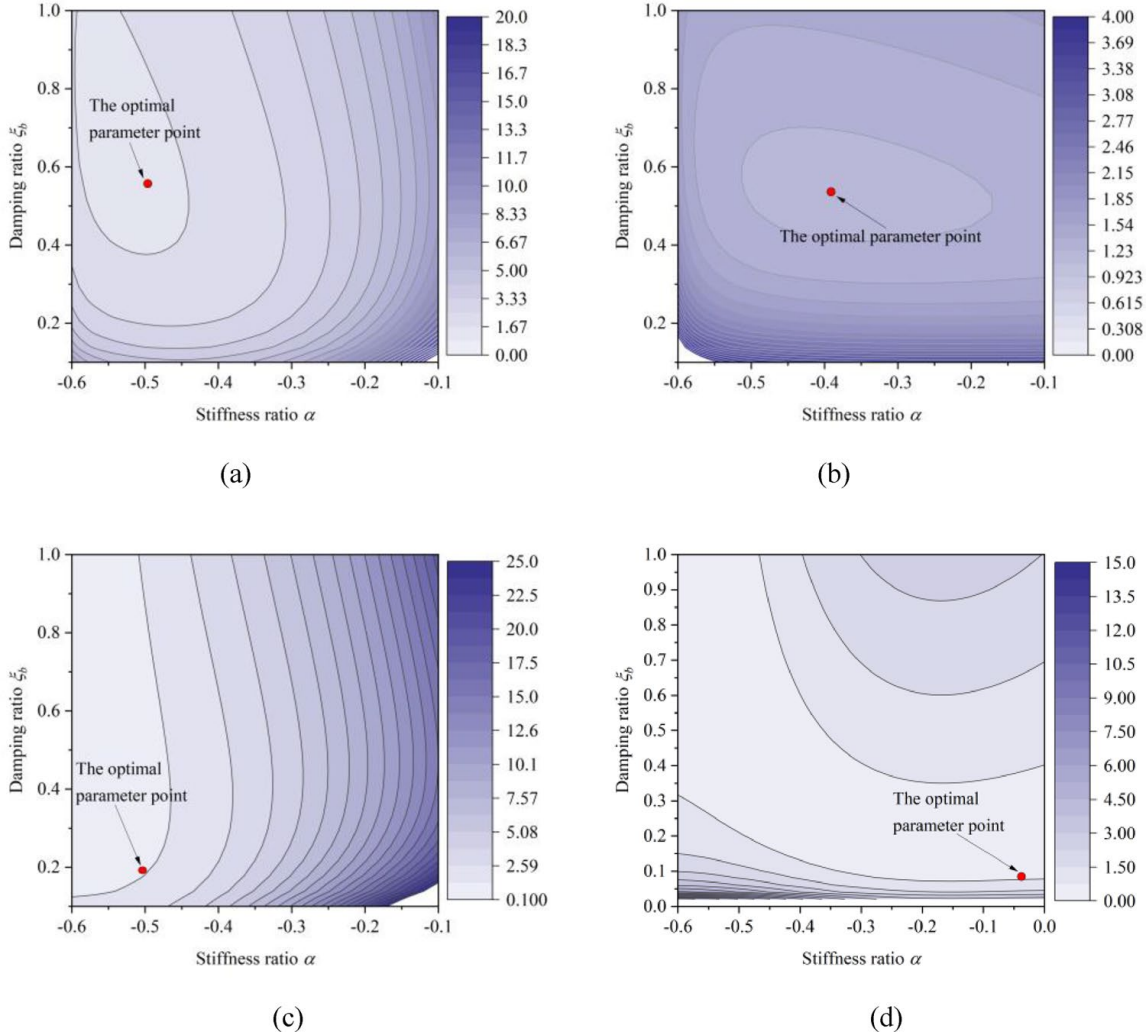
Contour plots of negative stiffness ratio $\alpha$ and damping ratio ${\xi }_{b}$ (${\mu }_{l}=4$, ${\mu }_{b}=0.1$, $\theta =1$, $\beta =0.3$). (**a**) Left building of the system based on NSID-1. (**b**) Right building of the system based on NSID-1. (**c**) Left building of the system based on NSID-2. (**d**) Right building of the system based on NSID-2

The contour plots of the performance index functions ${{\text{PI}}}_{i}^{{\text{NSID}}-1}$ and ${{\text{PI}}}_{i}^{{\text{NSID}}-2}$ can be obtained by using the Monte Carlo pattern search method as shown in Fig. 3. The trend of the contour plot can reflect the influence of the design parameter changes on the performance index function, and a satisfactory optimized value of the design parameter can be obtained from it.

The results of design parameter optimization for the vibration control system based on NSID-1 and NSID-2 are shown in Table 1. The optimized values of the design parameters for both vibration control systems have different characteristics and similarities. For the left building structure, both vibration control systems require a high negative stiffness ratio. In contrast, the values of the negative stiffness ratios for the parameter optimization of the right building structure are smaller. It should be noted that the parameter obtained by the search method is the better value in a certain region. Observing Fig. 3d, it can be found that the design parameters can be taken in a wide range of values so that ${{\text{PI}}}_{r}^{{\text{NSID}}-2}$ obtains a smaller value. In conclusion, the NSID-1 based vibration control system requires a higher damping ratio in order to optimize the parameters of both the left and the right building to achieve a better value of the performance index function. The vibration control system based on NSID-2, on the contrast, can achieve smaller values of the performance index function at lower damping ratios.

**Table 1:** The results of design parameter optimization for the vibration control systems.

Design parameters	System based on NSID-1		System based on NSID-2	
	${{\text{PI}}}_{l}^{{\text{NSID}}-1}$	${{\text{PI}}}_{r}^{{\text{NSID}}-1}$	${{\text{PI}}}_{l}^{{\text{NSID}}-2}$	${{\text{PI}}}_{r}^{{\text{NSID}}-2}$
${\alpha }_{{\text{opt}}}$	-0.493	-0.387	-0.505	-0.047
${{\xi }_{b}}_{{\text{opt}}}$	0.561	0.537	0.191	0.078

## Parameter optimization and analysis of vibration control systems

In this section, the relationship between the frequency response function and the system parameters of two vibration control systems is analyzed. The control variable method is used to investigate the effect of the system parameters on the frequency response function by assuming that the parameters of the uncontrolled system and the vibration control systems based on NSID-1 and NSID-2 are essentially the same.

### Effect of mass ratio ${{\varvec{\upmu}}}_{\mathbf{l}}$ on the frequency response function

**Figure 4:**
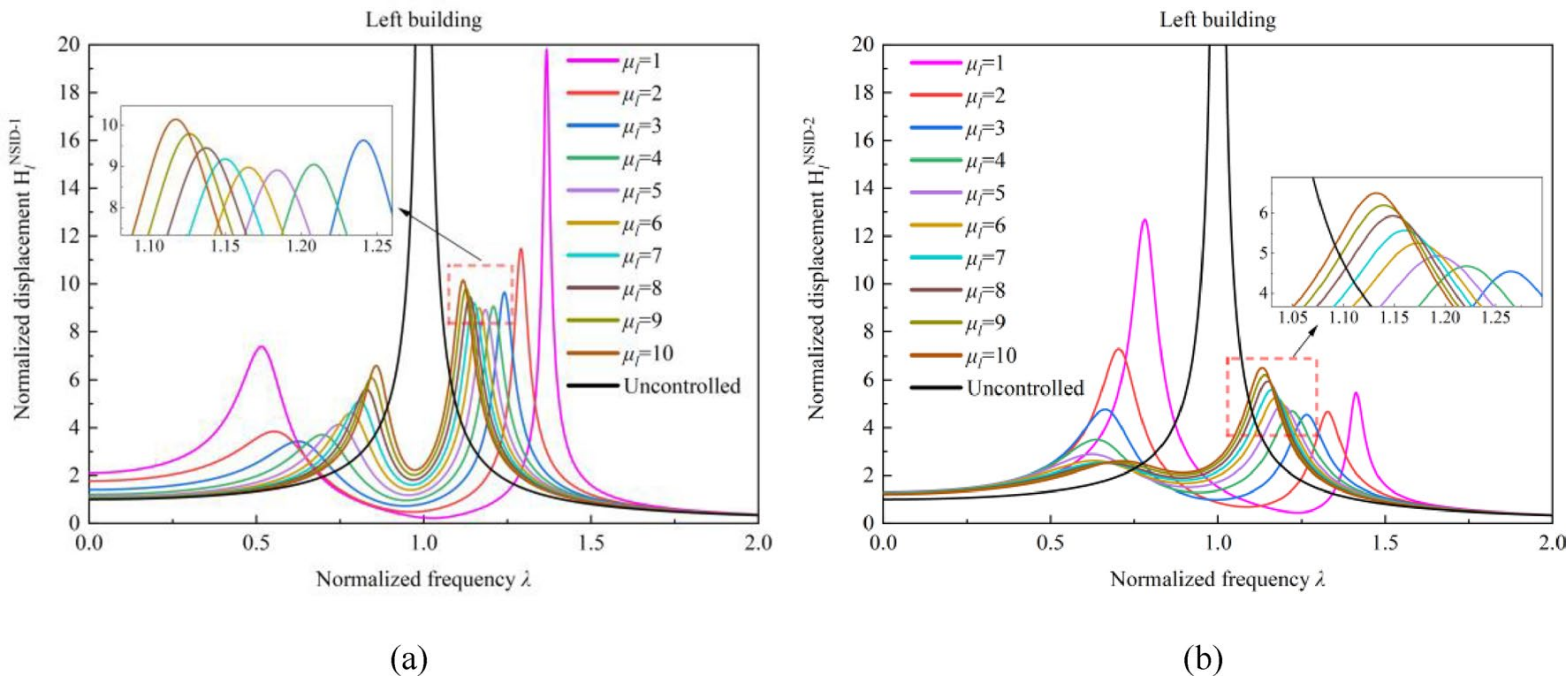
Effect of mass ratio *μ*_*l*_ variation on the frequency response function of the system. (**a**) System based on NSID-1. (**b**) System based on NSID-2

**Figure 5:**
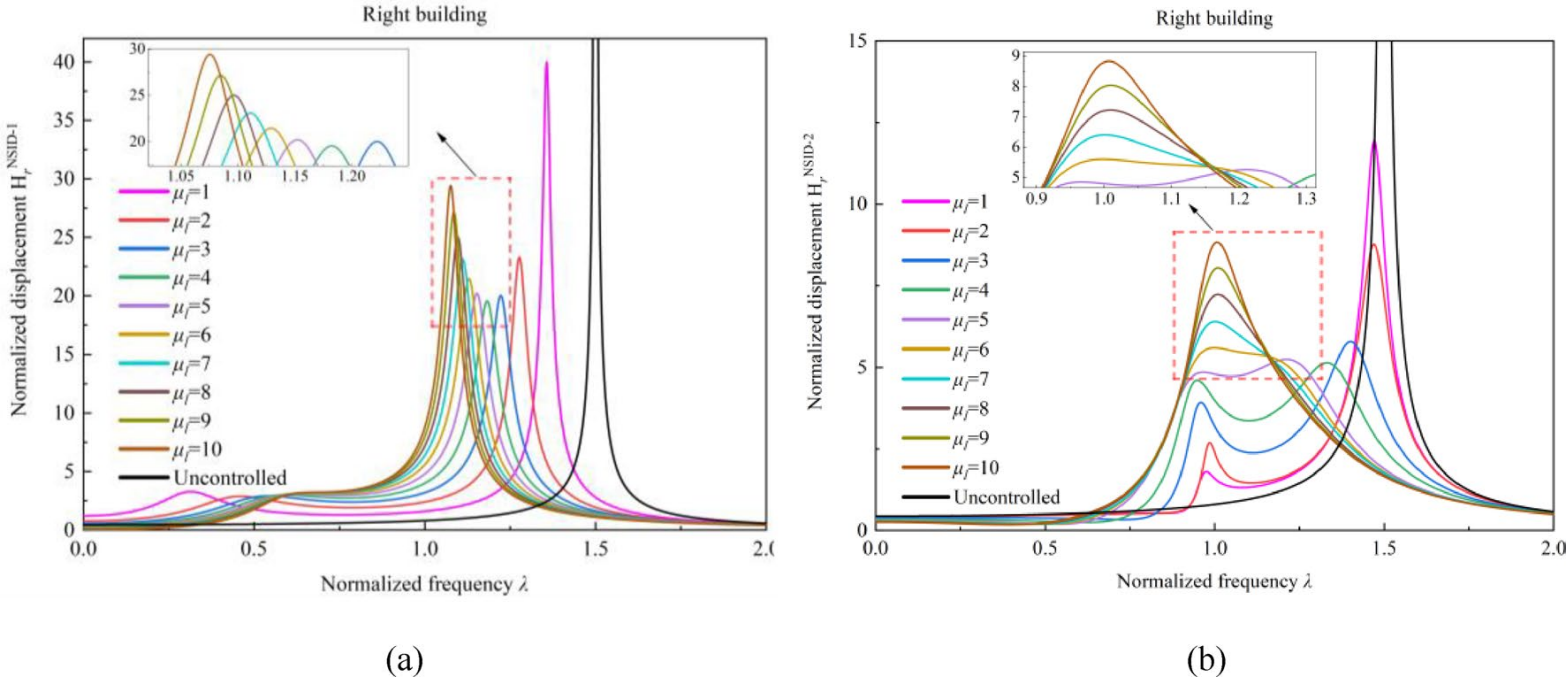
Effect of variation in mass ratio*μ*_*l*_ on the frequency response function of the system. (**a**) System based on NSID-1. (**b**) System based on NSID-2

Figures 4 and 5 reflect the effect of the mass ratio *μ*_*l*_ of adjacent building structures on the frequency response function of the control system. Among them, the black line is the frequency response function of the uncontrolled adjacent building structure. In the vibration control system based on NSID-1, the peak value of the frequency response function ${{\text{H}}}_{l}^{{\text{NSID}}-1}$ decreases by 42.08%, 15.99%, 6.08%, and 1.53% with the increase of mass ratio *μ*_*l*_ when the mass ratio $1 \le \mu_{l} \le 5$.When the mass ratio *μ*_*l*_ increases from 5 to 10, the peak value of the frequency response function ${{\text{H}}}_{l}^{{\text{NSID}}-1}$ increases by about 13.17%.In the vibration control system based on NSID-2, the peak value of the frequency response function ${{\text{H}}}_{l}^{{\text{NSID}}-2}$ decreases by 42.63%, 34.64%, 1.56% with the increase of mass ratio *μ*_*l*_ at $1 \le \mu_{l} \le 4$. The peak value of the frequency response function ${{\text{H}}}_{l}^{{\text{NSID}}-2}$ increases by about 33.66% when the mass ratio *μ*_*l*_ increases from 4 to 10.

In Fig. 5, the peak value of frequency response function ${{\text{H}}}_{r}^{{\text{NSID}}-1}$ decreases with the growth of mass ratio ${\mu }_{l}$ by 41.79%, 14.04%, and 2.32% as the mass ratio ${\mu }_{l}$ ($1{\le \mu }_{l}\le 4$) increases, and the peak value of frequency response function ${{\text{H}}}_{r}^{{\text{NSID}}-2}$ decreases by 26.6%, 34%, and 11.06%, in that order. The peak values of the frequency response function ${{\text{H}}}_{r}^{{\text{NSID}}-1}$ and ${{\text{H}}}_{r}^{{\text{NSID}}-2}$ show positive correlation with the mass ratio ${\mu }_{l}$ (${4\le \mu }_{l}\le 10$). Obviously, with the growth of the mass ratio ${\mu }_{l}$, the trend of the peak values of the frequency response functions ${{\text{H}}}_{r}^{{\text{NSID}}-1}$ and ${{\text{H}}}_{r}^{{\text{NSID}}-2}$ are both decreasing and then increasing. Overall, both ${{\text{H}}}_{r}^{{\text{NSID}}-1}$ and ${{\text{H}}}_{r}^{{\text{NSID}}-2}$ are more sensitive to the change of mass ratio ${\mu }_{l}$ for mass ratio ${\mu }_{l}\le 4$, and the vibration control system based on NSID-2 is more sensitive to the change of mass ratio ${\mu }_{l}$ for mass ratio ${\mu }_{l}\ge 4$. In addition, the frequency response function of the right building structure is more affected by the variation of mass ratio ${\mu }_{l}$ than that of the left building structure.

### Effect of stiffness ratio ${\varvec{\theta}}$ on the frequency response function of the system

**Figure 6:**
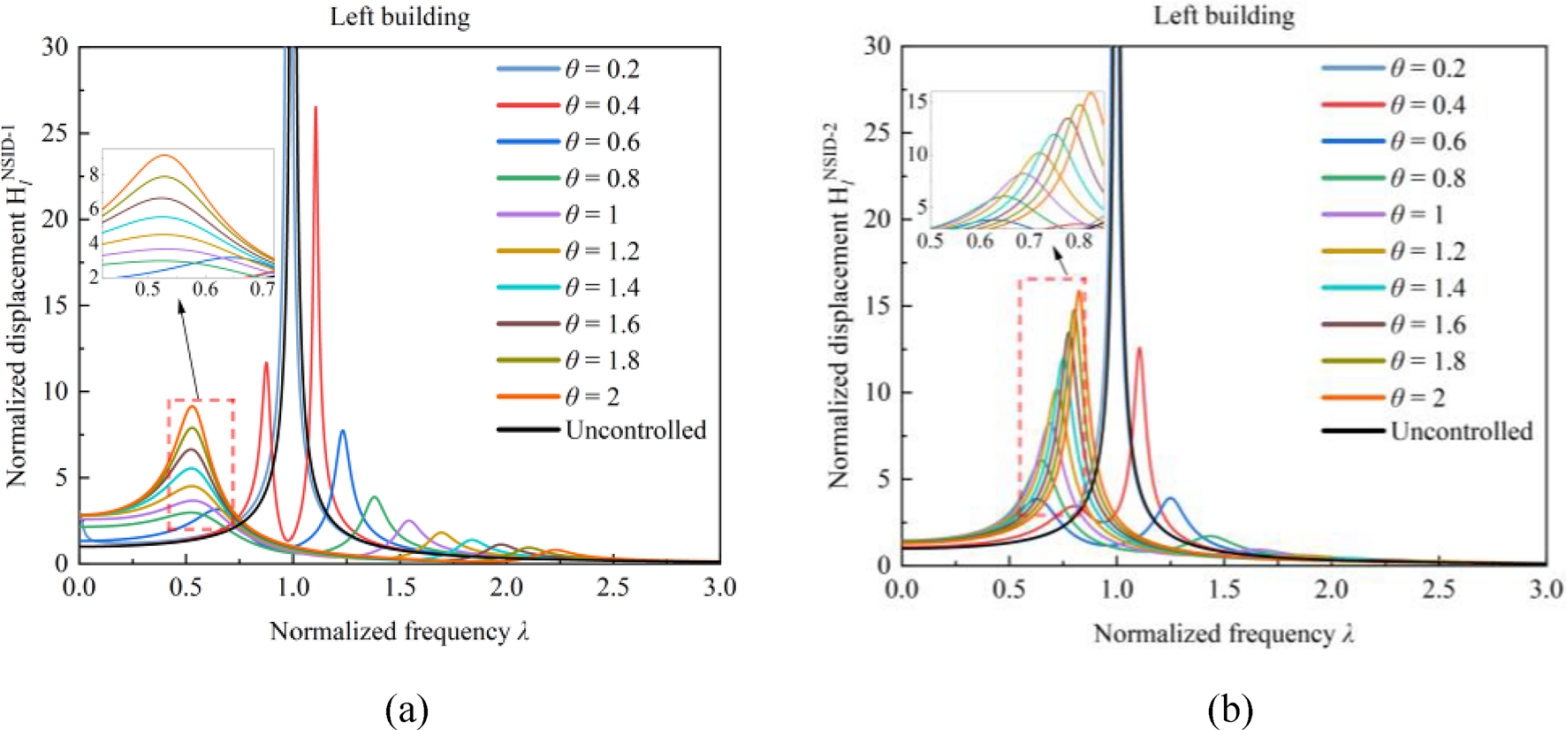
Effect of stiffness ratio *θ* on the frequency response function of the system. (**a**) System based on NSID-1. (**b**) System based on NSID-2

**Figure 7:**
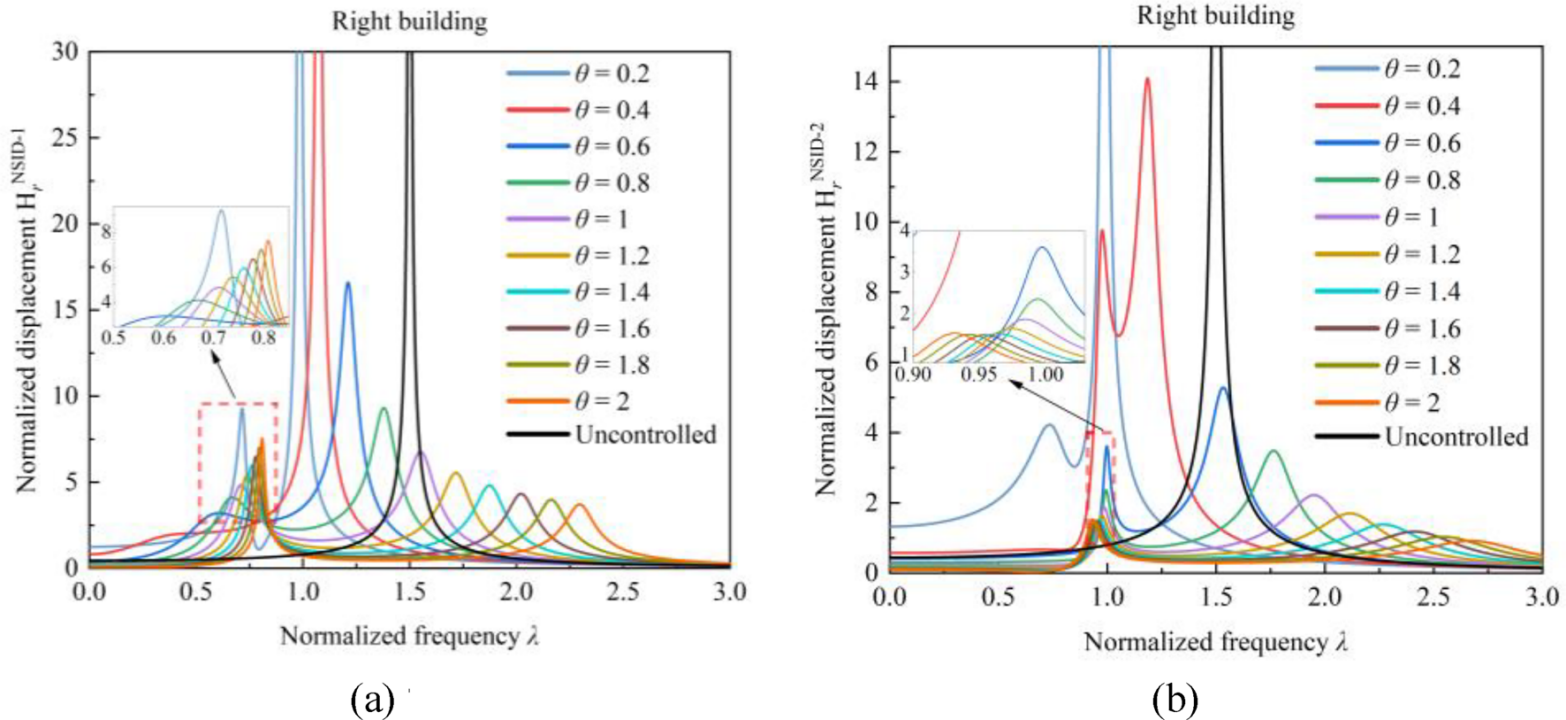
Effect of stiffness ratio *θ* on the frequency response function of the system. (**a**) System based on NSID-1. (**b**) System based on NSID-2

When the mass ratio ${\mu }_{l}=4$, the effects of the stiffness ratio *θ* on the system frequency response function are further analyzed to obtain Figs. 6 and 7. With the increase of the stiffness ratio *θ*, the peaks of the frequency response functions of both vibration control systems show the characteristic of decreasing and then increasing. In addition, the trends of the two peaks of the frequency response function in the figures have the same characteristics. When the stiffness ratio *θ* is small, the peak value of the frequency response function is the peak of the right waveform, and the corresponding frequency ratio *λ* is near 1. As the stiffness ratio *θ* increases, the peak value of the frequency response function gradually decreases, while the corresponding frequency ratio *λ* gradually increases. When the stiffness ratio *θ* increases to a certain extent, the peak value of the frequency response function takes the value of the left peak, and the corresponding frequency ratio *λ*$<1$. In general, the peak value of the frequency response function changes trend in the stiffness ratio *θ* to obtain a specific value of the turning point, so as to obtain a smaller value. With a stiffness ratio *θ* of 1, the frequency response functions of the building structures in Figs. 6 and 7 both achieve satisfactory peak values and facilitate the subsequent analysis of other parameters.

**Figure 8:**
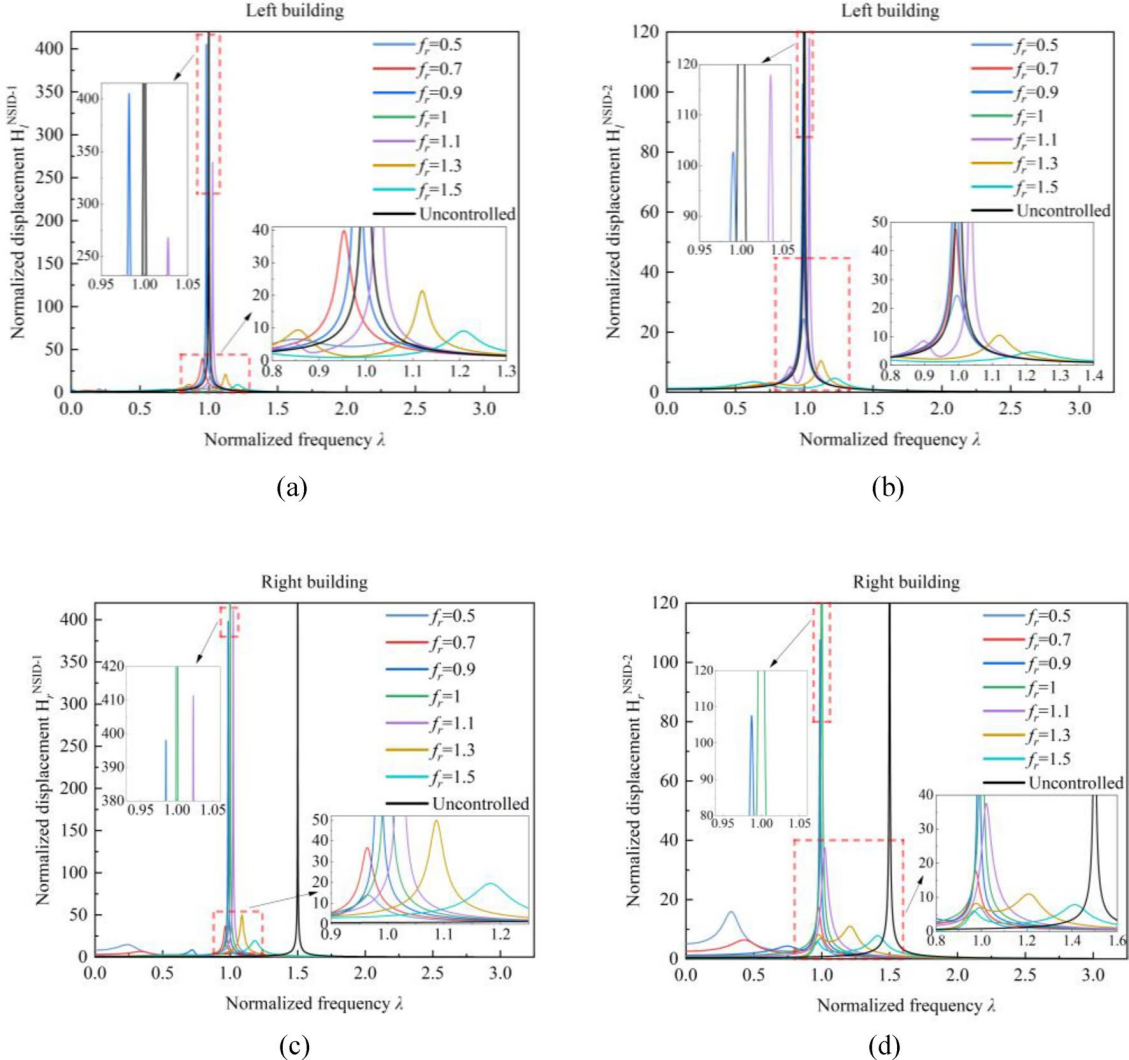
Effect of frequency ratio *f*_*r*_ on the system frequency response function. (**a**) System based on NSID-1. (**b**) System based on NSID-2. (**c**) System based on NSID-1. (**d**) System based on NSID-2

The frequency response function of the vibration control system increases significantly at both $\theta =0.2$ and $\theta =0.4$. Through the calculation of Eq. (6), it is found that the value of the frequency ratio ${f}_{r}$ is affected by the stiffness ratio $\theta$. Thus, the relationship between the frequency ratio ${f}_{r}$ and the frequency response function is analyzed to obtain Fig. 8. It is clear that the peak value of the frequency response function gradually increases as the frequency ratio ${f}_{r}$ is close to 1, and the incremental magnitude of the peak value of the frequency response function is significantly increased. This phenomenon is more obvious in the vibration control system based on NSID-1 than in the vibration control system based on NSID-2. In particular, when ${f}_{r}=1$, the frequency response function curve of the system almost coincides with that of the uncontrolled system, resulting in a system with poor vibration control performance.

### Effect of stiffness ratio ${\varvec{\beta}}$ on the frequency response function of the system

**Figure 9:**
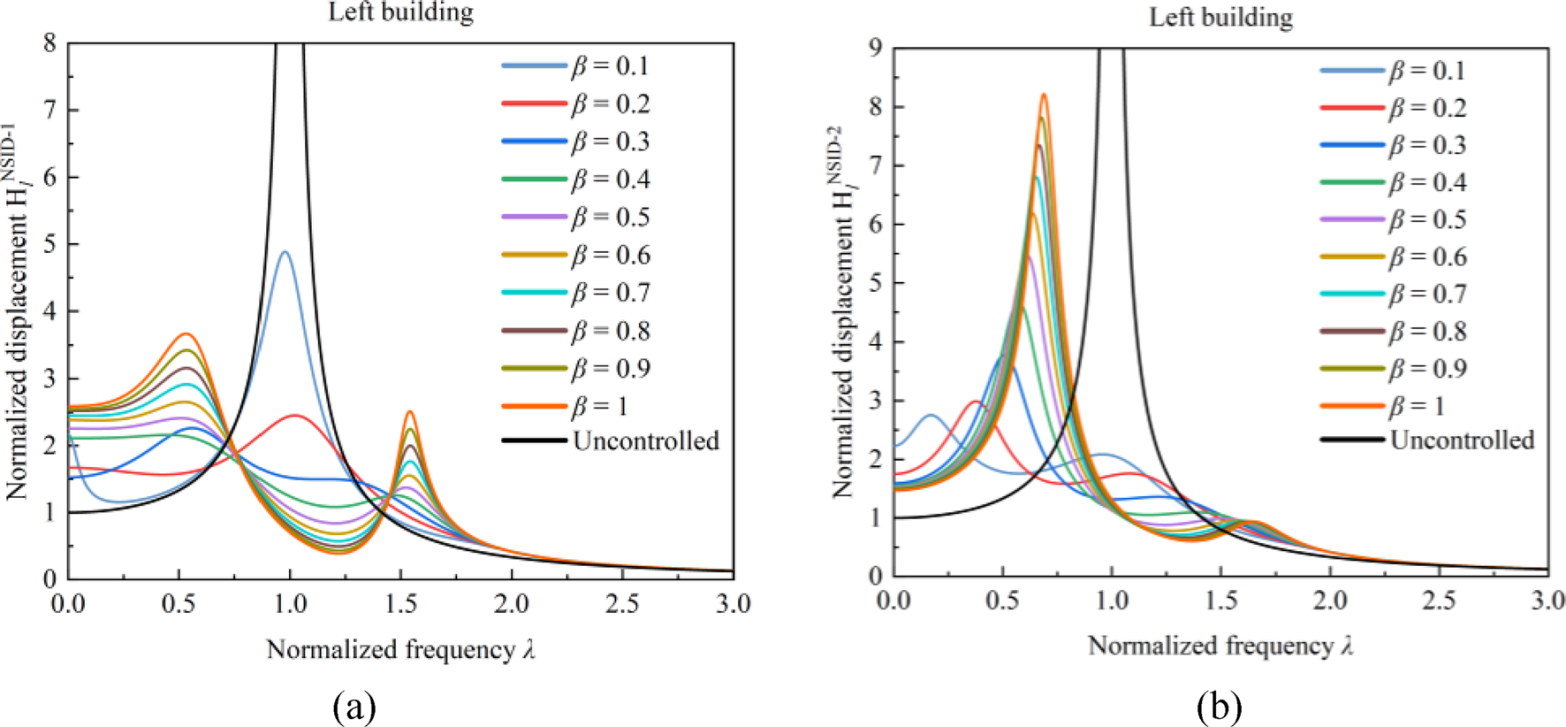
Effect of stiffness ratio ${\varvec{\beta}}$ on the frequency response function of the system. (**a**) System based on NSID-1. (**b**) System based on NSID-2

**Figure 10:**
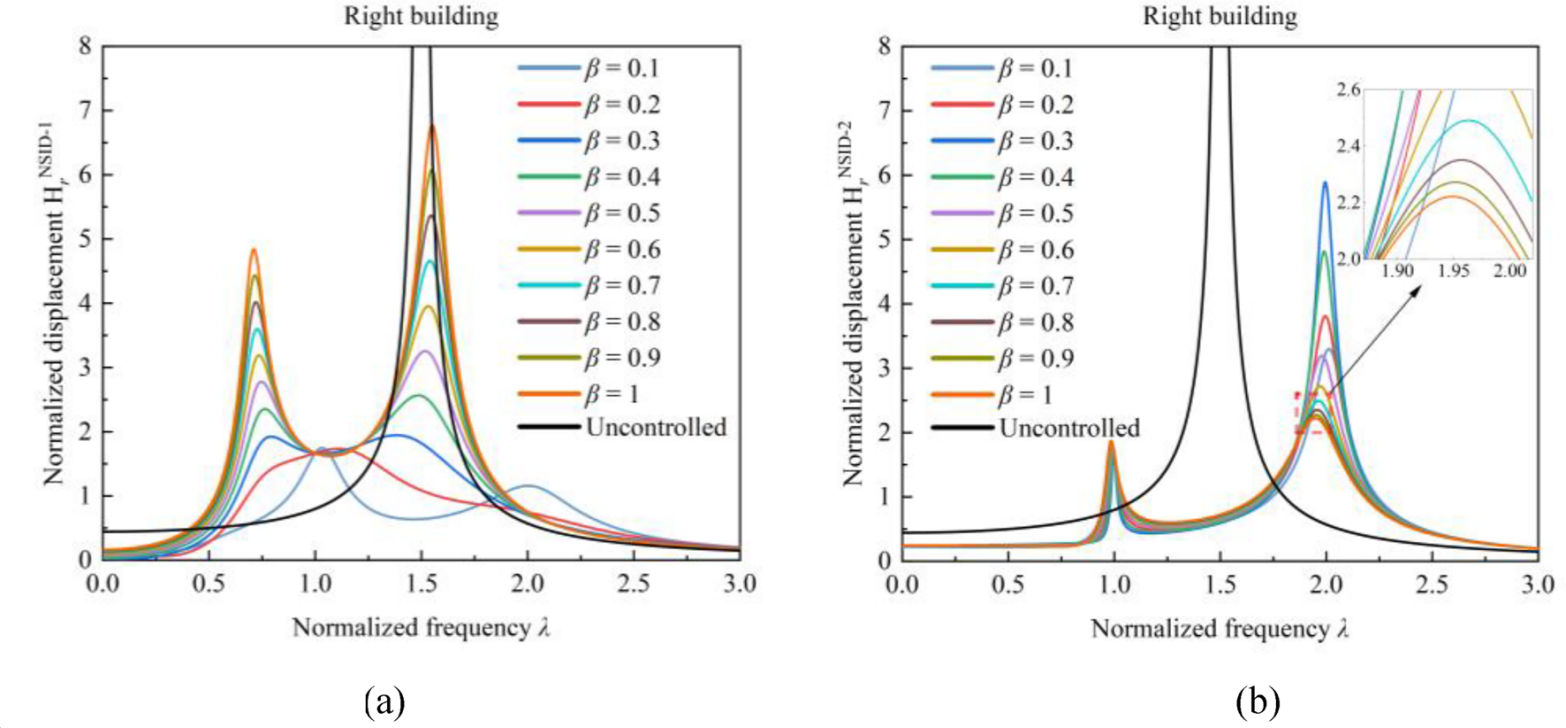
Effect of stiffness ratio ${\varvec{\beta}}$ on the frequency response function of the system. (**a**) System based on NSID-1. (**b**) System based on NSID-2

With the increase of stiffness ratio ${\varvec{\beta}}$, the peak value of frequency response function ${\mathbf{H}}_{{\varvec{l}}}^{\mathbf{N}\mathbf{S}\mathbf{I}\mathbf{D}-2}$ of the vibration control system based on NSID-2 gradually increases, while the peak value of frequency response function ${\mathbf{H}}_{{\varvec{r}}}^{\mathbf{N}\mathbf{S}\mathbf{I}\mathbf{D}-2}$ first increases and then decreases. The peak values of the frequency response functions of the vibration control system based on NSID-1 all exhibit the characteristic of decreasing first and then increasing. Observing Figs. 9 and 10, it can be found that the frequency response function of the system is more sensitive to the change of stiffness ratio ${\varvec{\beta}}$ when the stiffness ratio ${\varvec{\beta}}$ is small. For the frequency response functions of different building structures in different systems, the values of the stiffness ratios ${\varvec{\beta}}$ that cause significant changes in the peak value of the frequency response function are different. This phenomenon is particularly obvious when the stiffness ratio ${\varvec{\beta}}$ increases from 0.1 to 0.2 in Fig. 9a, and the peak value of the frequency response function ${\mathbf{H}}_{{\varvec{l}}}^{\mathbf{N}\mathbf{S}\mathbf{I}\mathbf{D}-1}$ decreases by about 49.86%. When the stiffness ratio ${\varvec{\beta}}$ is increased from 0.3 to 0.4, the frequency response function ${\mathbf{H}}_{{\varvec{r}}}^{\mathbf{N}\mathbf{S}\mathbf{I}\mathbf{D}-2}$ increases by about 33.68%. The peak value of the frequency response function is minimized when the stiffness ratio ${\varvec{\beta}}$ is obtained as 1 in Fig. 10b, but the peak values of the other frequency response functions are higher. Considering the magnitude of variation in the values of several frequency response functions, taking the stiffness ratio ${\varvec{\beta}}=0.3$ makes the system relatively more robust, which makes it easier to analyse the effect of the inerter ratio ${{\varvec{\mu}}}_{{\varvec{b}}}$ on the system.

### Effect of inerter mass ratio ${{\varvec{\mu}}}_{{\varvec{b}}}$ on the system frequency response function

**Figure 11:**
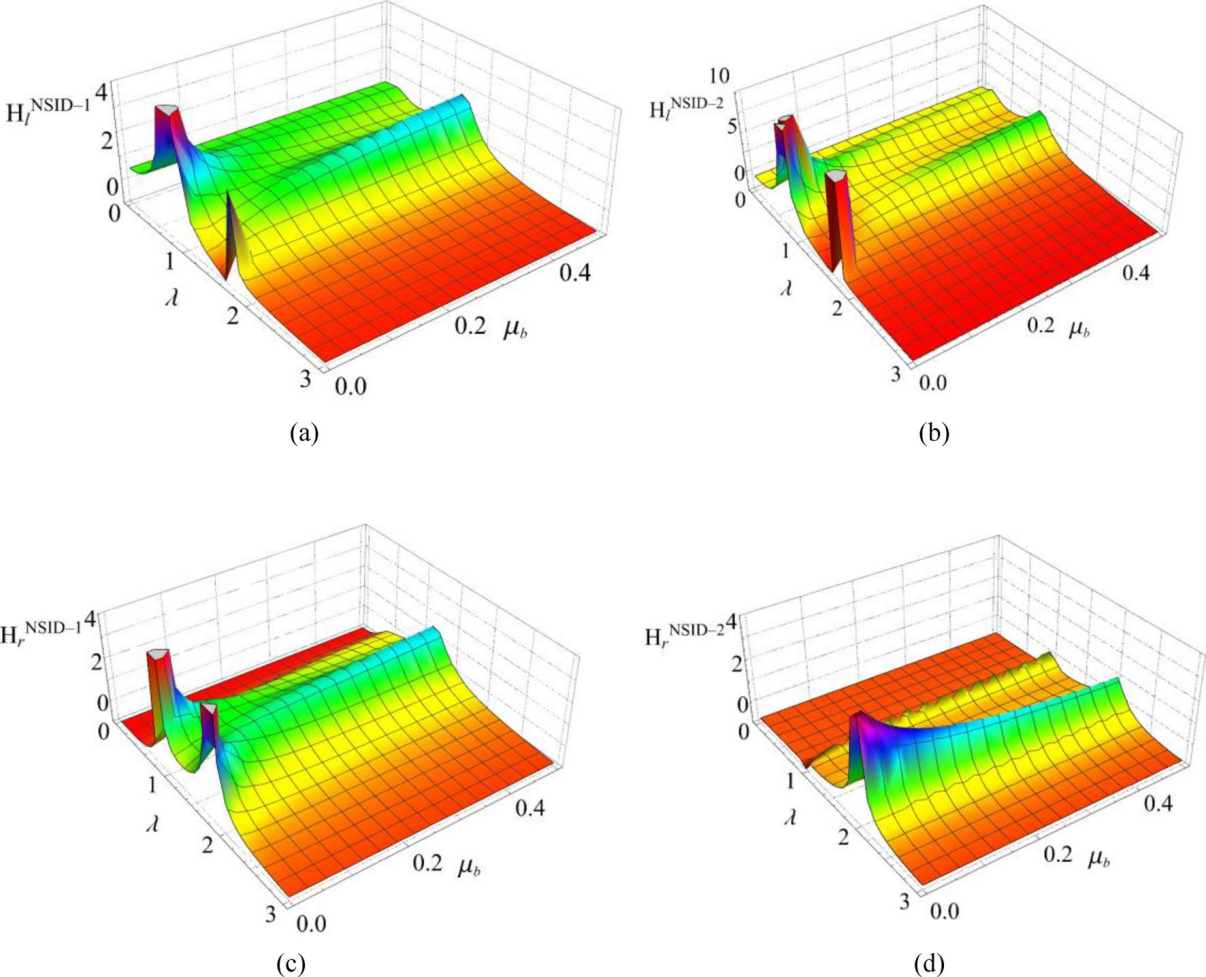
Three-dimensional plots of the influence of the mass ratio ${{\varvec{\mu}}}_{{\varvec{b}}}$ on the frequency response function of the system. (**a**) Left building of system based on NSID-1. (**b**) Left building of system based on NSID-2. (**c**) Right building of system based on NSID-1. (**d**) Right building of system based on NSID-2

After analyzing several system parameters, more satisfactory values of the system frequency response function were determined. In order to determine the parameter ${\mu }_{b}$, a three-dimensional plot as shown in Fig. 11 was drawn in order to clearly reflect the influence of the inerter mass ratio ${\mu }_{b}$ on the robustness of the two vibration control systems. The peak value of the frequency response function ${{\text{H}}}_{l}^{{\text{NSID}}-1}$ decreases and then increases with the increase of the inerter mass ratio ${\mu }_{b}$ in Fig. 11a, and the optimum value corresponds to the inerter mass ratio ${\mu }_{b}$ located near 0.1, but the peak value of the frequency response function ${{\text{H}}}_{l}^{{\text{NSID}}-2}$ continues to decrease with the increase of the inerter mass ratio ${\mu }_{b}$ in Fig. 11d. When the inerter mass ratio ${\mu }_{b}$ is close to 0, the peak values of the frequency response functions of both vibration control systems based on NSID-1 and NSID-2 are significantly increased. When the inerter mass ratio ${\mu }_{b}$ is small, the increase of inerter mass ratio ${\mu }_{b}$ can effectively reduce the peak value of the frequency response function. However, the frequency response function of the system increases slightly (except for ${{\text{H}}}_{r}^{{\text{NSID}}-2}$) when the inerter mass ratio ${\mu }_{b}$ is larger than a certain value. Observing Fig. 11, it can be found that the curve change of the system frequency response function starts to flatten when the inerter mass ratio ${\mu }_{b}\ge 0.3$. Considering the influence of the increase of the inerter mass ratio ${\mu }_{b}$ on the peak value of the system frequency response function, the selection of the inerter mass ratio ${\mu }_{b}=0.1$ can make the system obtain better stability and robustness.

## Time domain simulation

### Two single-degree-of-freedom structures

In this section, four real seismic excitation records and two artificial waves are selected as shown in Table 2. Displacement time histories of adjacent single-degree-of-freedom (SDOF) building structures under seismic excitations are obtained by loading seismic waves to compare the vibration control performance of vibration control system based on NSID-1 and NSID-2. The design parameters of the control device can be obtained by referring to the system parameter analysis and optimization process in Section "Parameter optimization and analysis of vibration control systems", and the specific parameter value settings of the vibration control systems in MATLAB are given in Table 3. The natural frequencies of the left and right building structures in the uncontrolled state are 6.229 Hz and 4.963 Hz, respectively.

**Table 2:** Real seismic records and artificial waves.

Input motion name	Location	Recording station	Maximum acceleration (g)	Year
Chi-Chi	Taiwan	TCU045	0.349	September 20, 1999
Imperial_Valley	USA	USGS STATION 5115	0.3152	October 15, 1979
Landers	USA	000 SCE STATION 24	0.7803	June 28, 1992
Kocaeli	Turkey	YARIMCA(KOERI330)	0.361	August 17, 1999
RGB1	–	–	0.402	–
RGB2	–	–	0.462	–

**Table 3:** The parameters table of the system based on NSID-1 and NSID-2.

Parameters		${m}_{i}$(or b) (kg)	${c}_{i}$(or ${{\text{c}}}_{{\text{d}}}$) (N∙s/m)	${{\varvec{k}}}_{{\varvec{i}}}$(or ${{\varvec{k}}}_{{\varvec{p}}}$) (N/m)	${{\varvec{k}}}_{{\varvec{n}}{\varvec{s}}}$(N/m)
Left Building	$4.0\times {10}^{5}$	$1.0\times {10}^{5}$	$6.127\times {10}^{8}$	–	
Right Building	$3.5\times {10}^{5}$	$1.0\times {10}^{5}$	$3.404\times {10}^{8}$	–	
LB	NSID-1	$4.0\times {10}^{4}$	$1.520\times {10}^{6}$	$1.021\times {10}^{8}$	$-4.361\times {10}^{7}$
	NSID-2		$0.909\times {10}^{6}$		$-4.606\times {10}^{7}$
RB	NSID-1	$4.0\times {10}^{4}$	$1.116\times {10}^{6}$	$1.021\times {10}^{8}$	$-4.861\times {10}^{7}$
	NSID-2		$1.188\times {10}^{6}$		$-4.912\times {10}^{7}$

In this table, LB and RB denote the optimizing left building structure and right building structure, respectively.

**Figure 12:**
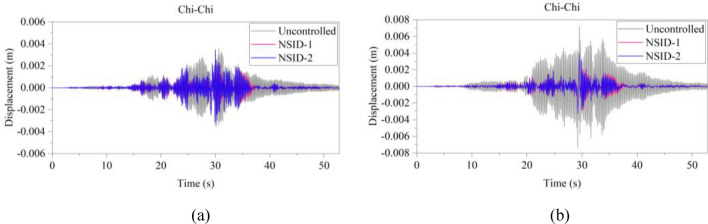
Comparison of displacement time histories of adjacent building structures under Chi-Chi seismic excitation. (**a**) Left building structure. (**b**) Right building structure

**Figure 13:**
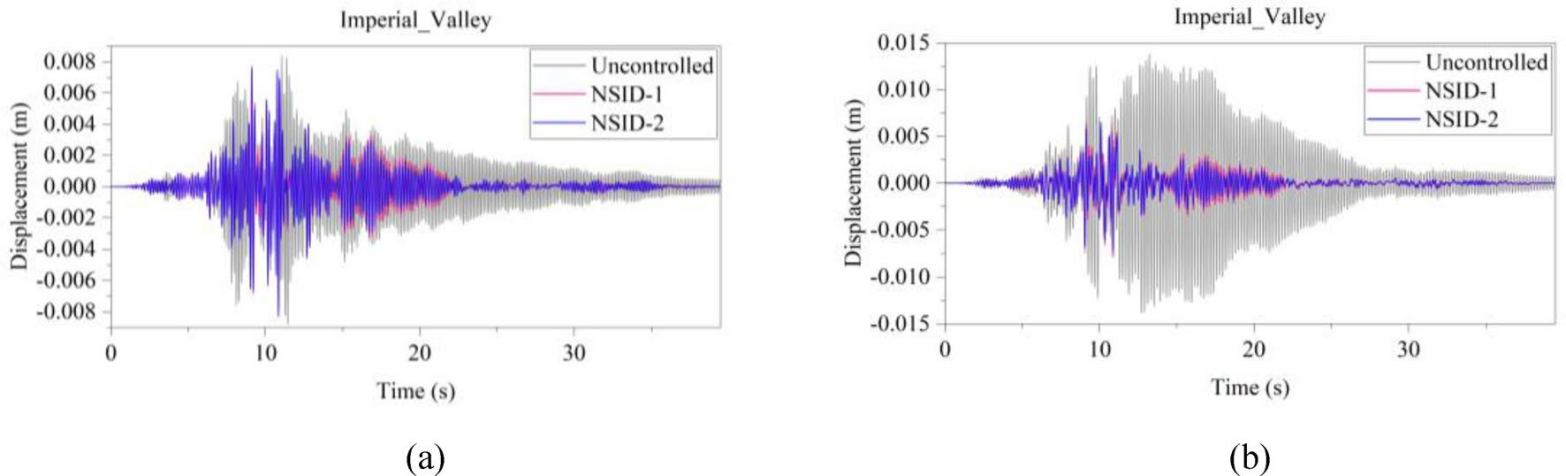
Comparison of displacement time histories of adjacent building structures under Imperial_Valley seismic excitation. (**a**) Left building structure. (**b**) Right building structure

**Figure 14:**
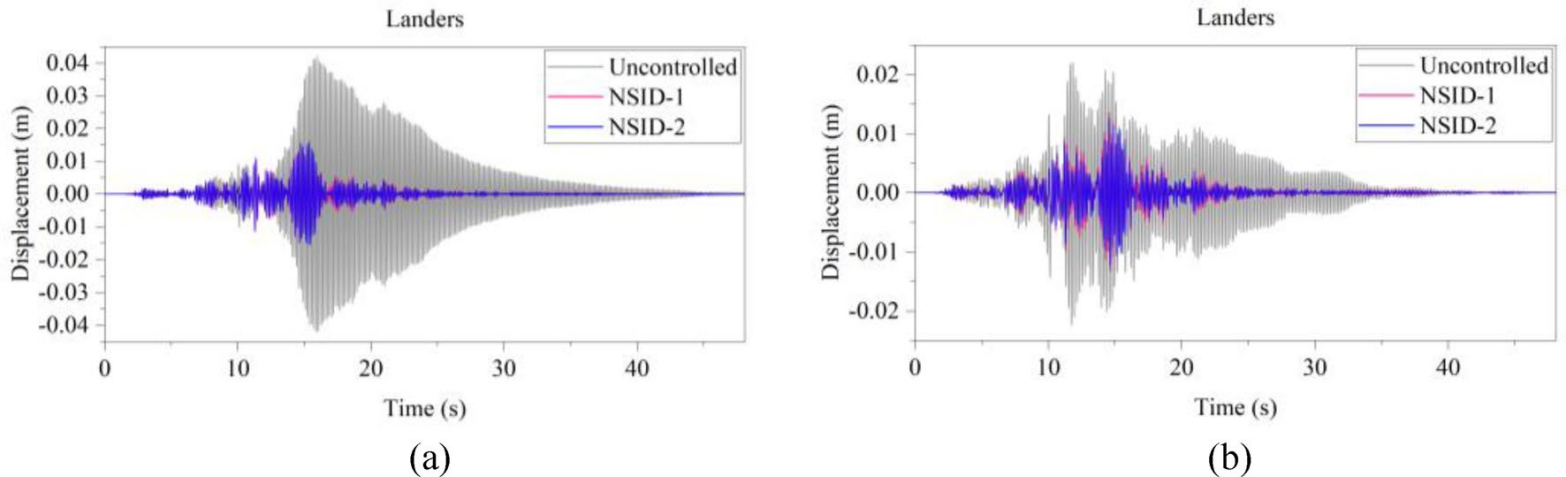
Comparison of displacement time histories of adjacent building structures under Landers seismic excitation. (**a**) Left building structure. (**b**) Right building structure

**Figure 15:**
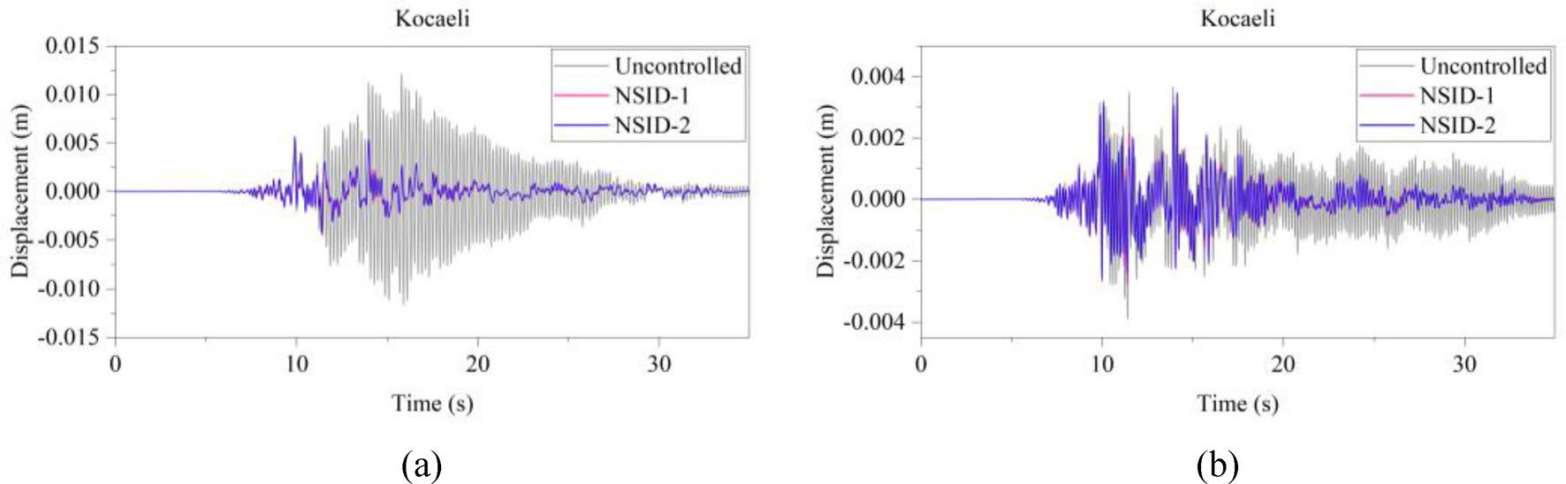
Comparison of displacement time histories of adjacent building structures under Kocaeli seismic excitation. (**a**) Left building structure. (**b**) Right building structure

**Figure 16:**
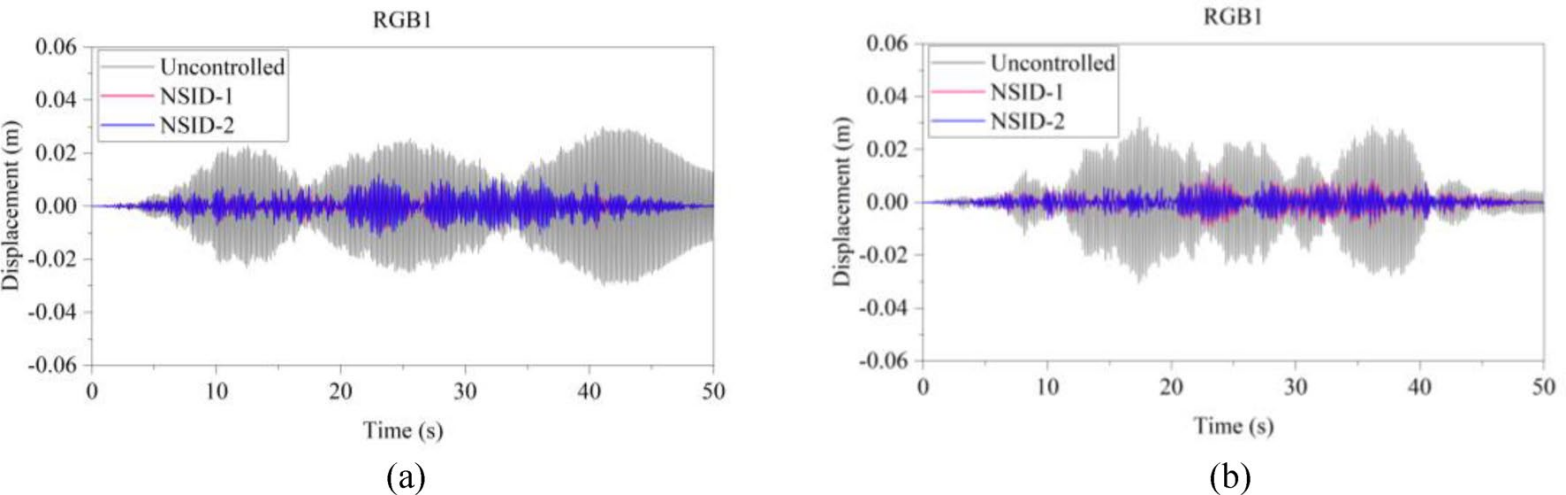
Comparison of displacement time histories of adjacent building structures under RGB1 wave excitation. (**a**) Left building structure. (**b**) Right building structure

**Figure 17:**
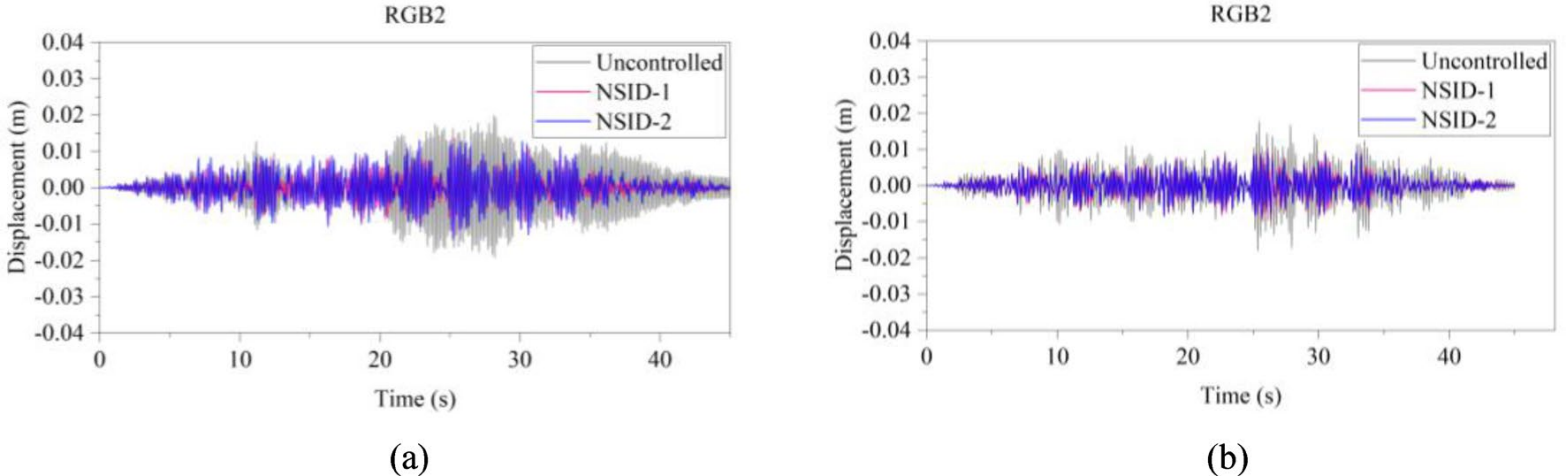
Comparison of displacement time histories of adjacent building structures under RGB2 wave excitation. (**a**) Left building structure. (**b**) Right building structure

The response results of the two vibration control systems and the uncontrolled system under multiple seismic excitations are shown in Figs. 12, 13, 14, 15, 16 and 17. It is clear that both NSID-1 and NSID-2 as the connecting structure of the adjacent building structures can effectively reduce the displacement response amplitude of the two building structures, which improves the seismic capacity of the building structures.

The peak and root mean square values of the displacements time histories of the vibration control system and no control system under seismic excitation have been recorded in detail in Tables 4 and 5. It is obvious that both NSID-1 and NSID-2 are able to reduce the peak displacement response of the adjacent building structures, thus reducing the damage caused by seismic actions on the building structures. In particular, under strong earthquake Landers, the NSID-1-based vibration control system reduces the peak displacement of the left building structure and the right building structure by about 63.43% and 39.39%, respectively; and the vibration control system based on NSID-2 reduces the peak displacement of the left building structure and the right building structure by about 62.35% and 43.73%, respectively. Under the action of artificial waves, NSID-1 reduces the peak displacement of building structure by at least 38.082%, and NSID-2 reduces the peak response by at least 36.565%. For the control effect on the root mean square of peak displacement, NSID-1 is at least 34.301% and at most 77.517%; NSID-2 is at least 34.037% and at most 78.109%. Overall, both vibration control systems can effectively reduce the displacement amplitude of the adjacent building structure and improve the stability and reliability of the adjacent building structure. It should be noted that there are differences in the natural frequencies of the left and right building structures in the two vibration control systems, and the displacement frequency response function curves of the building structures also show variation amplitude with the change of frequency ratio ${\varvec{\lambda}}$, which leads to the different vibration control performance of the systems when they are subjected to the excitation of different seismic waves.

**Table 4:** The peak values of displacement time histories of the adjacent building structures under different seismic excitation.

Earthquake	Left building (mm)			Right building (mm)		
	NSID-1	NSID-2	UC	NSID-1	NSID-2	UC
Chi-Chi	3.522 (2.05)	3.477 (3.29)	3.596	3.805 (48.64)	3.864 (47.84)	7.409
Imperial_Valley	8.078 (8.35)	8.310 (5.72)	8.815	7.881 (42.97)	7.118 (48.49)	13.819
Landers	15.400 (63.43)	15.855 (62.35)	42.123	13.562 (39.39)	12.590 (43.73)	22.379
Kocaeli	3.458 (11.44)	3.471 (11.09)	3.905	5.481 (54.75)	5.688 (53.05)	12.116
RGB1	11.693 (61.253)	12.230 (59.474)	30.178	11.647 (63.937)	9.891 (69.374)	32.296
RGB2	13.885 (30.117)	14.159 (28.738)	19.869	10.283 (42.684)	9.134 (49.089)	17.941

In this table, (1) the “#” in (#) indicates the percentage decrease in the peak displacement of the vibration control system based on NSID-1 and NSID-2 compared to the peak displacement of the uncontrolled system; (2) UC represents uncontrolled system; (3) # =(UC-NSID-1 or NSID-2)∗100%/UC.

**Table 5:** Root mean square (RMS) of displacement time histories of the adjacent building structures under different seismic excitation.

Earthquake	Left building (mm)			Right building (mm)		
	NSID-1	NSID-2	UC	NSID-1	NSID-2	UC
Chi-Chi	0.498 (34.301)	0.500 (34.037)	0.758	0.470 (69.755)	0.427 (72.523)	1.554
Imperial_Valley	1.160 (35.805)	1.130 (37.465)	1.807	1.121 (73.698)	1.007 (76.373)	4.262
Landers	2.345 (77.517)	3.079 (70.479)	10.430	1.979 (60.229)	1.779 (64.248)	4.976
Kocaeli	0.558 (34.813)	0.554 (35.280)	0.856	0.780 (74.367)	0.786 (74.170)	3.043
RGB1	3.106 (75.382)	3.389 (73.139)	12.617	2.877 (73.494)	2.376 (78.109)	10.854
RGB2	3.327 (40.916)	3.572 (36.565)	5.631	2.595 (38.082)	2.385 (43.092)	4.191

In this table, (1) the “#” in (#) indicates the percentage decrease in the root mean square displacements of the vibration control system based on NSID-1 and NSID-2 compared to the root mean square displacement of the uncontrolled system; (2) UC represents uncontrolled system; (3) # =(UC-NSID-1 or NSID-2)∗100%/UC.

**Figure 18:**
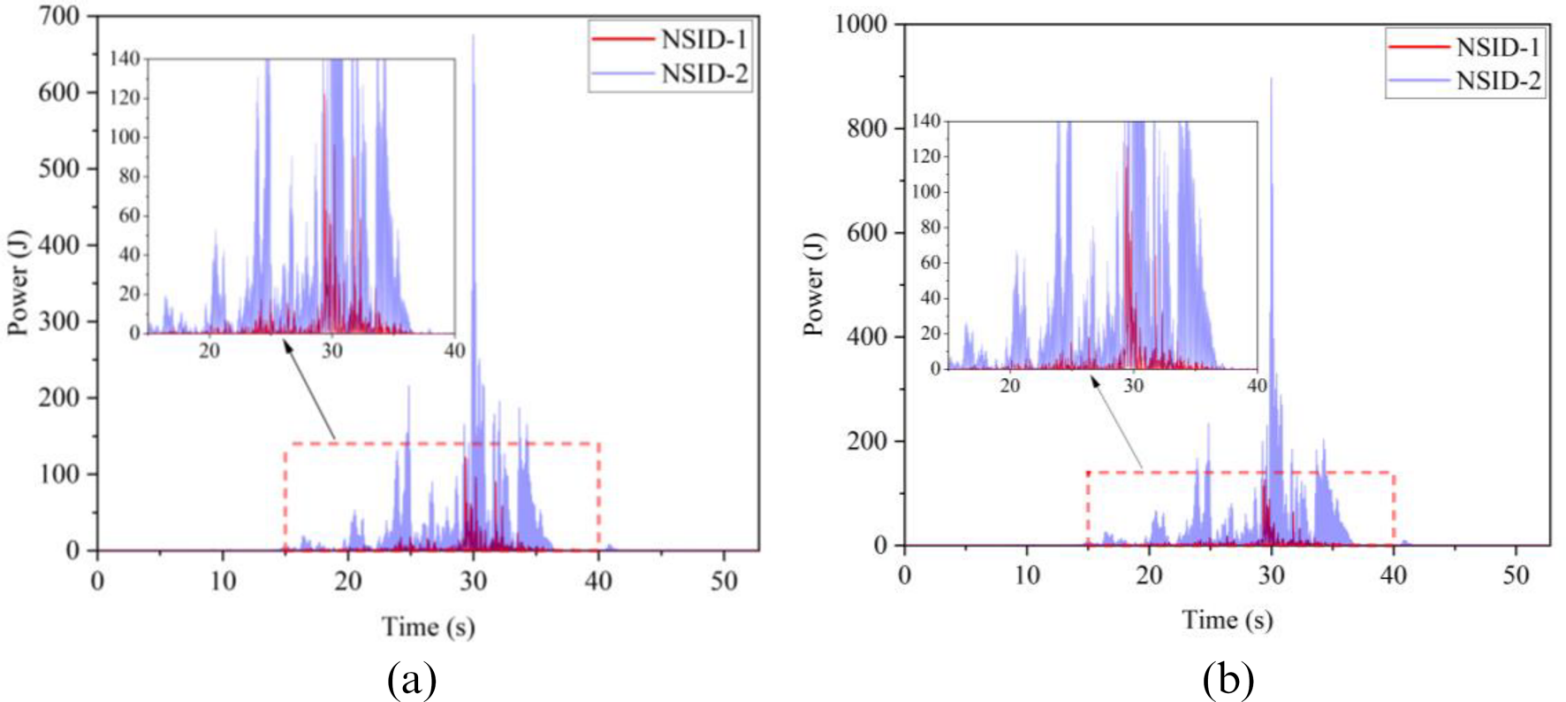
Kinetic energy of the control device under Chi-Chi seismic excitation. (**a**) Optimizing the left building structure. (**b**) Optimizing the right building structure

**Figure 19:**
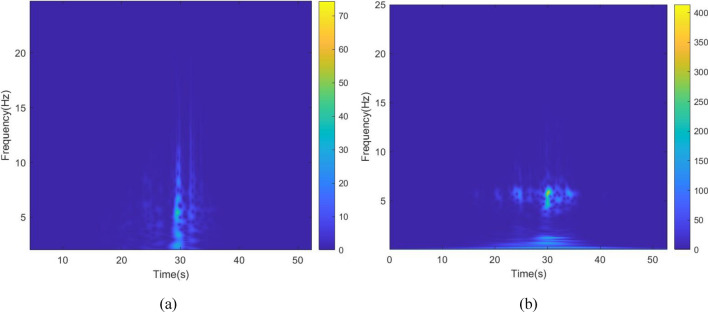
Time–frequency energy diagram of the inerter when optimizing the left building structure. (**a**) System based on NSID-1. (**b**) System based on NSID-2

**Figure 20:**
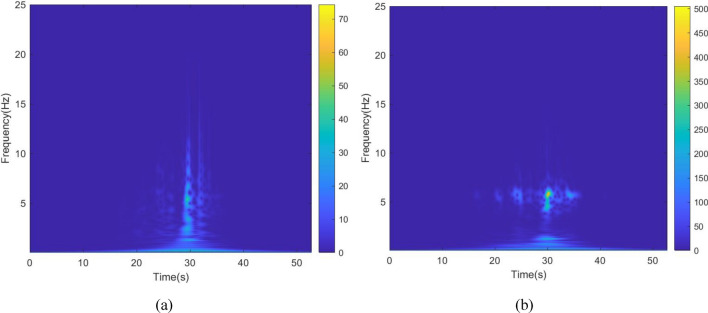
Time–frequency energy diagram of the inerter when optimizing the right building structure. (**a**) System based on NSID-1. (**b**) System based on NSID-2

The kinetic energy of the inerter in the vibration control system under Chi-Chi seismic excitation is shown in Fig. 18. The peak and RMS of kinetic energy of inerter under other seismic actions are shown in Table 6. The kinetic energy of the inerter in the vibration control system based on NSID-2 is significantly larger compared to that of the vibration control system based on NSID-1. This means that the vibration control system using NSID-1 to connect adjacent structures mainly consumes the energy generated by vibration through dampers, while the vibration control system using NSID-2 to connect adjacent structures mainly converts seismic energy into kinetic energy of inerter. Therefore, it would be more advantageous to use NSID-2 when considering energy harvesting in the vibration control system of adjacent structures. The kinetic energy distribution of the inerter in both vibration control systems versus the frequency and time of the seismic wave is shown in Figs. 19 and 20. The energy is mainly concentrated in the lower frequency region, while the peak energy is located near 5 Hz. Obviously, the peak and total amount of available kinetic energy is significantly higher in the vibration control system based on NSID-2.

**Table 6:** The peak and Root mean square (RMS) of Kinetic energy of the inerter.

Earthquake	Peak kinetic energy of inerter at optimizing the left building structure (J)		Peak kinetic energy of inerter at optimizing the right building structure (J)	
	NSID-1	NSID-2	NSID-1	NSID-2
Chi-Chi	122.324 (6.356)	675.716 (38.232)	126.284 (6.528)	897.167 (46.818)
Imperial_Valley	634.303 (40.622)	2724.750 (215.600)	678.602 (37.243)	3464.821 (264.393)
Landers	2123.791 (111.739)	12,276.702 (961.898)	1152.100 (82.566)	14,636.561 (1118.548)
Kocaeli	203.512 (18.621)	679.002 (51.234)	202.313 (19.426)	846.013 (60.984)
RGB1	586.665 (83.035)	7795.385 (1053.765)	447.466 (71.071)	7183.842 (955.105)
RGB2	1341.559 (148.885)	9587.730 (1273.874)	1079.580 (136.281)	10,373.774 (1277.698)

In this table, the “#” in (#) indicates the RMS of Kinetic energy of the inerter.

### Two multi-degree-of-freedom structures

**Figure 21:**
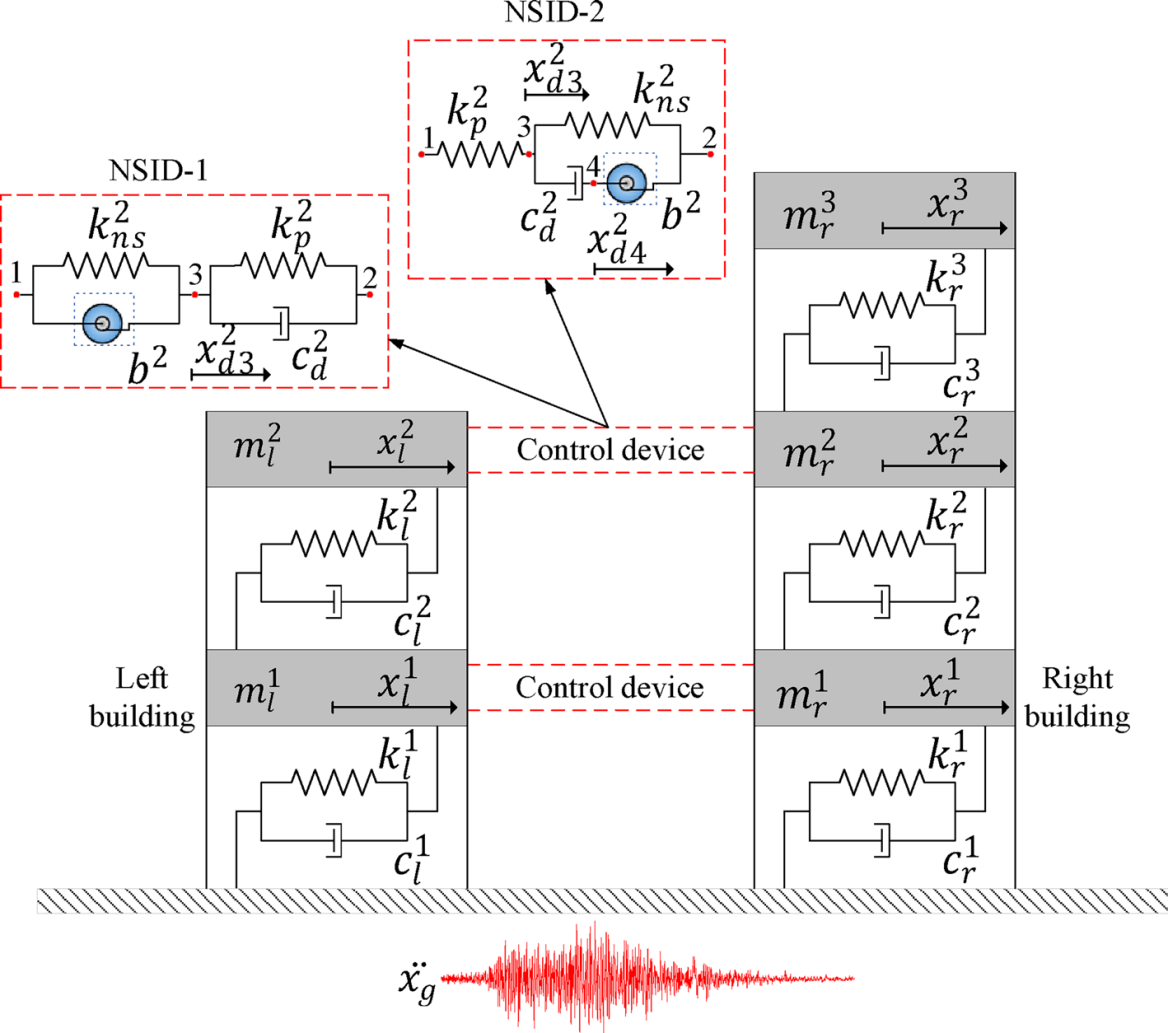
Simplified model of vibration control system for two MDOF building structures based on NSID-1 and NSID-2.

A simplified model of the vibration control system for two multi-degree-of-freedom (MDOF) structures is shown in Fig. 21.

The superscripts for the parameters in Fig. 21 indicate the number of stories. The specific parameter value settings of the vibration control systems in MATLAB are given in Table 7. In the uncontrolled state, the natural frequencies of the left building are 3.850 Hz and 10.079 Hz, while the natural frequencies of the right building are 2.209 Hz, 6.189 Hz and 8.944 Hz, respectively. The vibration control performance of vibration control systems based on NSID-1 and NSID-2 is compared by loading the seismic waves shown in Table 2, and obtaining the displacement time histories of adjacent MDOF building structures under seismic excitation.

**Table 7:** The parameters table of the two MDOF systems based on NSID-1 and NSID2.

Parameters		Story	${m}_{i}$(or b) (kg)	${c}_{i}$(or ${{\text{c}}}_{{\text{d}}}$) (N∙s/m)	${k}_{i}$(or ${k}_{p}$) (N/m)	${k}_{ns}$(N/m)
Left Building		1	$4.0\times {10}^{5}$	$1.0\times {10}^{5}$	$6.127\times {10}^{8}$	–
		2				
Right Building		1	$3.5\times {10}^{5}$	$1.0\times {10}^{5}$	$3.404\times {10}^{8}$	–
		2				
		3				
LB	NSID-1	1	$2.0\times {10}^{4}$	$3.289\times {10}^{5}$	$2.553\times {10}^{7}$	$-1.334\times {10}^{7}$
		2				
	NSID-2	1	$2.0\times {10}^{4}$	$2.026\times {10}^{5}$	$2.553\times {10}^{7}$	$-0.972\times {10}^{7}$
		2				
RB	NSID-1	1	$2.0\times {10}^{4}$	$2.457\times {10}^{5}$	$2.553\times {10}^{7}$	$-1.517\times {10}^{7}$
		2				
	NSID-2	1	$2.0\times {10}^{4}$	$2.609\times {10}^{5}$	$2.553\times {10}^{7}$	$-1.071\times {10}^{7}$
		2				

In this table, LB and RB denote the optimizing left building structure and right building structure, respectively.

**Figure 22:**
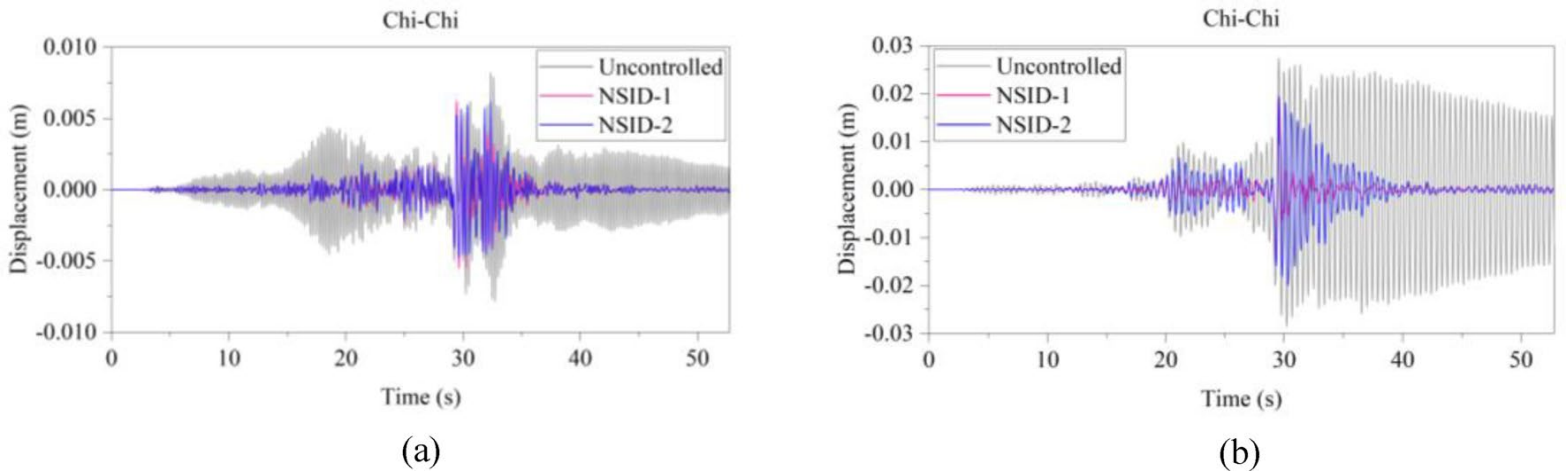
Comparison of displacement time histories of adjacent MDOF building structures under Chi-Chi seismic excitation. (**a**) Top floor of the left building structure. (**b**) Top floor of the right building structure

**Figure 23:**
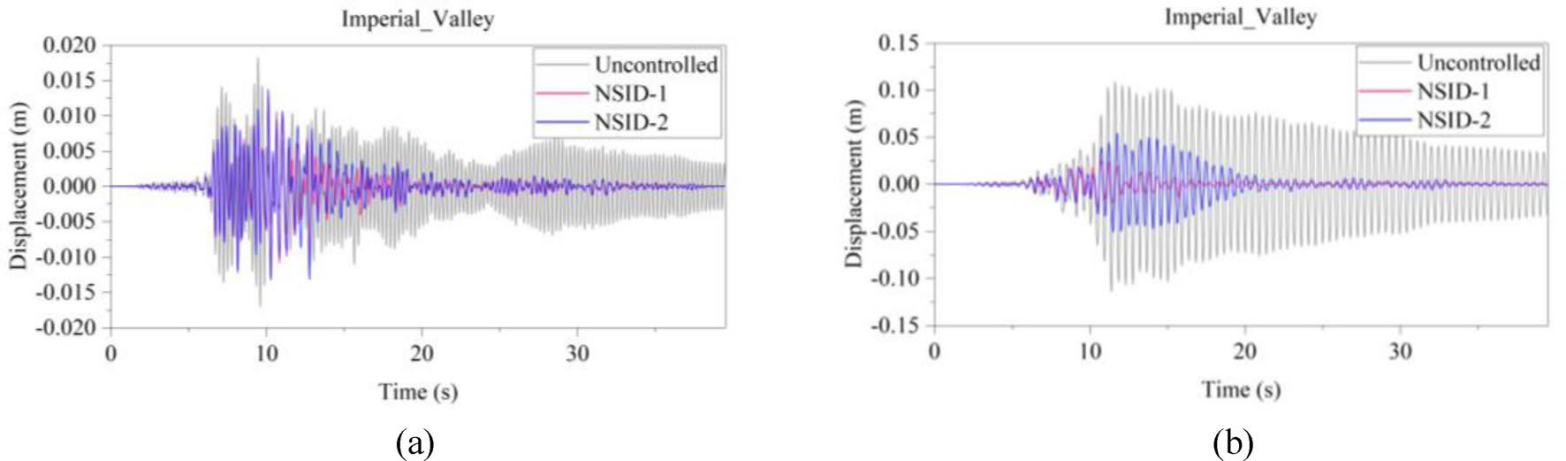
Comparison of displacement time histories of adjacent MDOF building structures under Imperial_Valley seismic excitation. (**a**) Top floor of the left building structure. (**b**) Top floor of the right building structure

**Figure 24:**
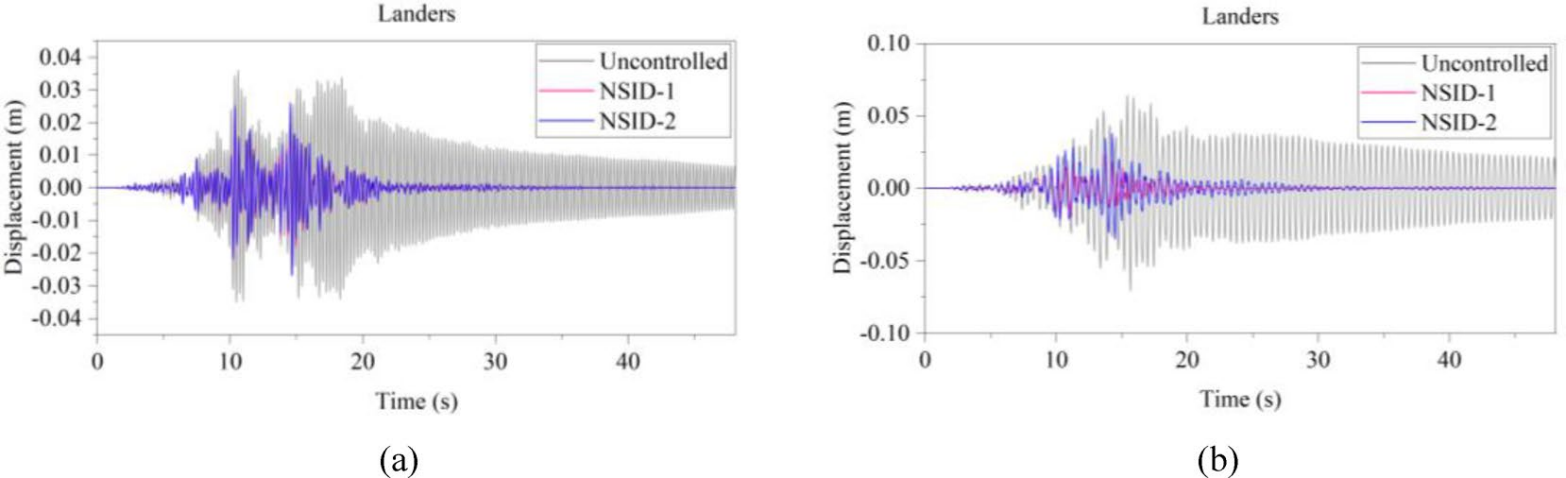
Comparison of displacement time histories of adjacent MDOF building structures under Landers seismic excitation. (**a**) Top floor of the left building structure. (**b**) Top floor of the right building structure

**Figure 25:**
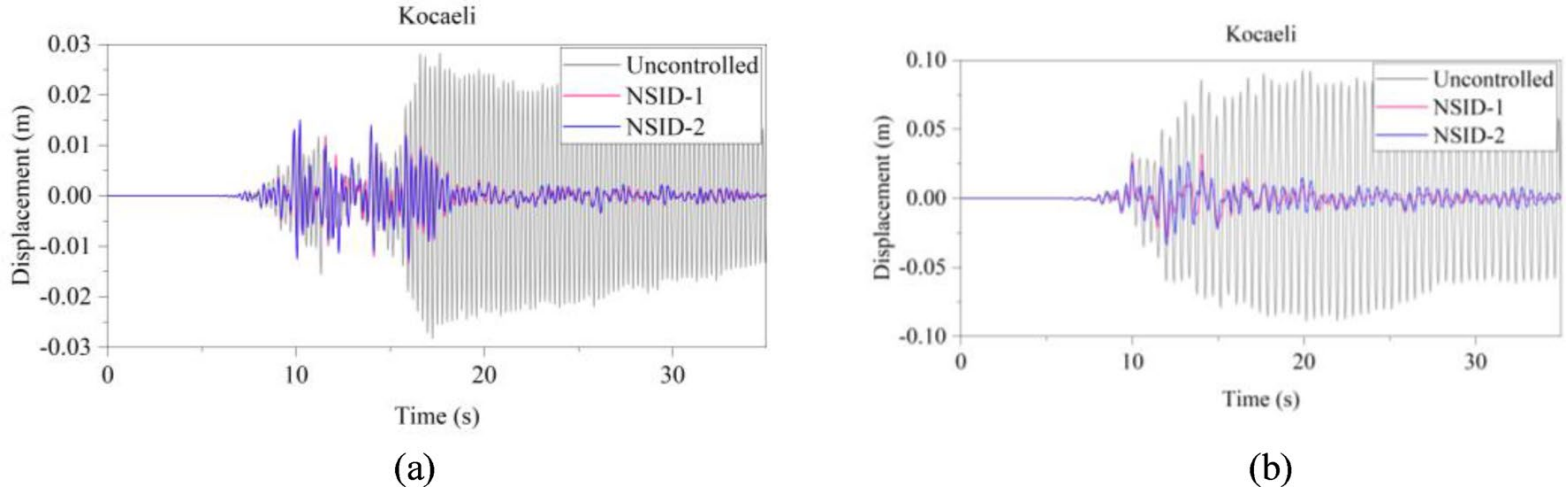
Comparison of displacement time histories of adjacent MDOF building structures under Kocaeli seismic excitation. (**a**) Top floor of the left building structure. (**b**) Top floor of the right building structure

**Figure 26:**
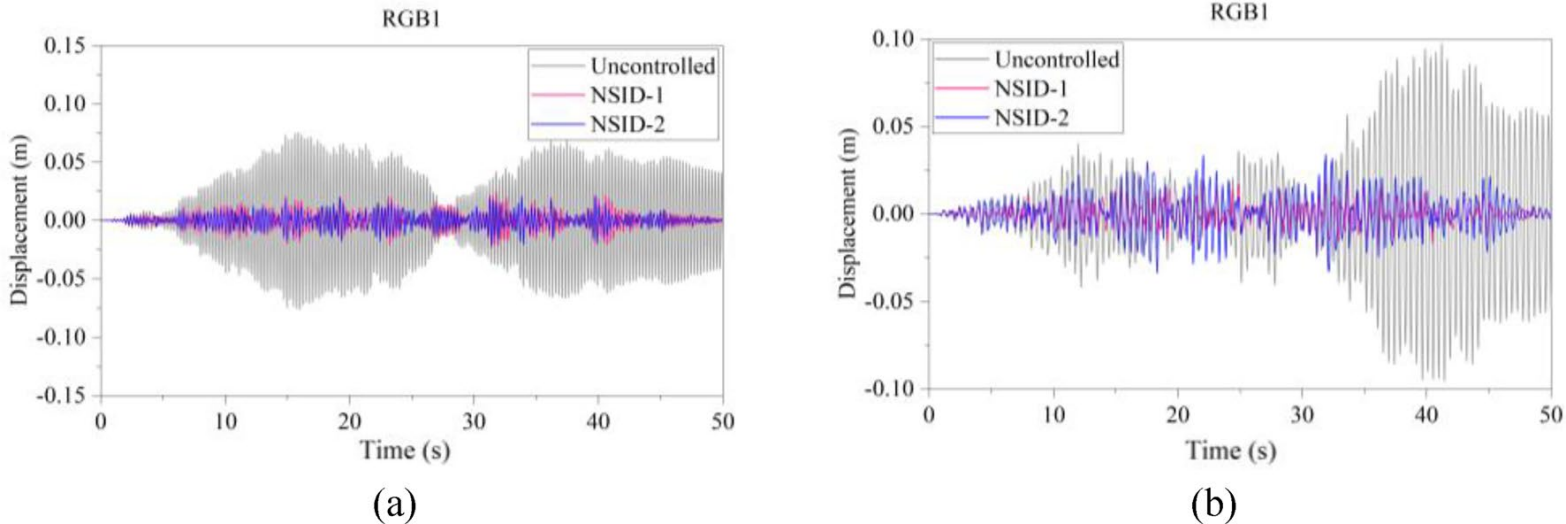
Comparison of displacement time histories of adjacent MDOF building structures under RGB1 wave excitation. (**a**) Top floor of the left building structure. (**b**) Top floor of the right building structure

**Figure 27:**
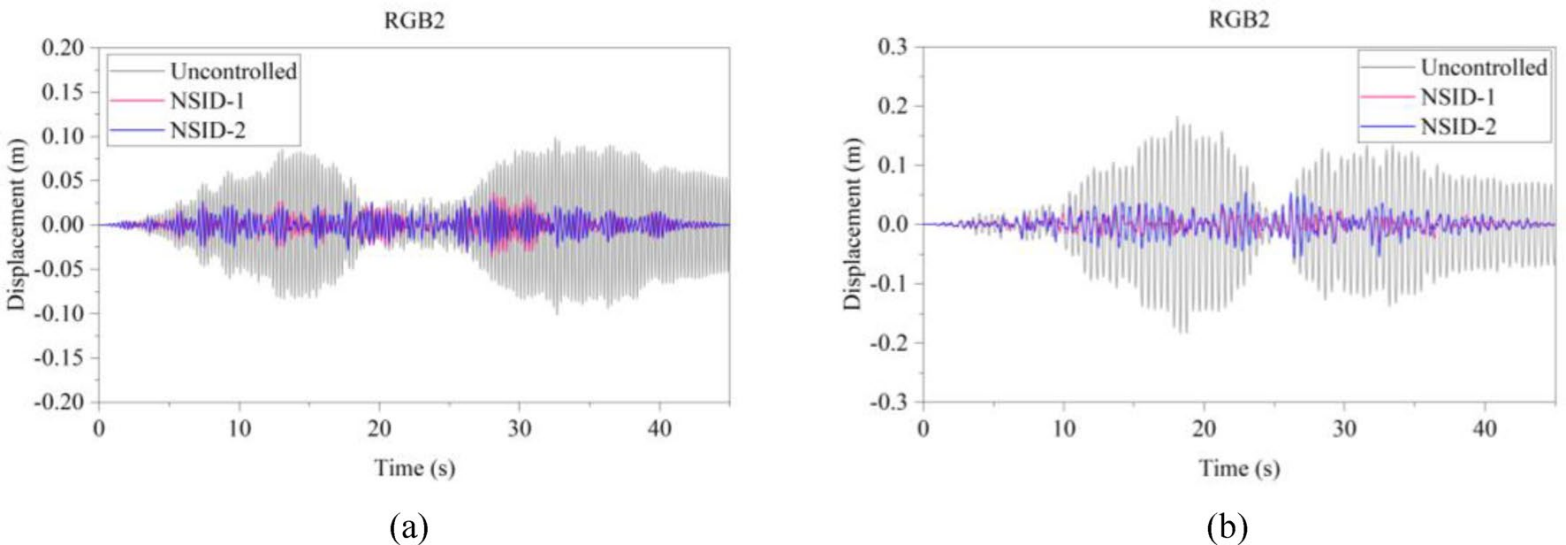
Comparison of displacement time histories of adjacent MDOF building structures under RGB2 wave excitation. (**a**) Top floor of the left building structure. (**b**) Top floor of the right building structure

The response results of the two MDOF structures under seismic excitation are shown in Figs. 22, 23, 24, 25, 26, 27 for the controlled and uncontrolled states. It is obvious that NSID-1 and NSID-2 can effectively reduce the displacement response amplitude of the adjacent MDOF building structures and improve the seismic capacity of the building structure.

The peak and root mean square values of the displacement time histories for the vibration control system and uncontrolled system under seismic excitation are detail in Tables 8 and 9. It is clear that both NSID-1 and NSID-2 are able to reduce the peak displacement response of the adjacent building structures, thus decreasing the structural damage caused by seismic actions. Among them, under the action of real seismic wave Kocaeli, the vibration control system based on NSID-1 reduces the peak displacement of the left building structure and the right building structure by about 49.903% and 65.560%, respectively; and the vibration control system based on NSID-2 reduces the peak displacement of the left building structure and the right building structure by about 46.750% and 63.892%, respectively. Under the action of artificial waves, NSID-1 reduces the peak displacement of building structure by at least 75.271%, and NSID-2 reduces the peak response by at least 67.104%. For the control effect on the root mean square of peak displacement, NSID-1 is at least 42.260% and at most 88.605%; NSID-2 is at least 41.653% and at most 84.666%. Overall, the two vibration control devices in the adjacent MDOF system are equally effective in reducing the displacement amplitude of the structures and improving the stability and reliability of the adjacent building structures. Similar to Section "Two single-degree-of-freedom structures", the natural frequencies of the two building structures in the system are different, thus leading to different vibration control performances of the two systems when subjected to different seismic waves.

**Table 8:** The peak displacement time histories of the top floors of the adjacent MDOF building structures under different seismic excitation.

Earthquake	Left Building (mm)			Right Building (mm)		
	NSID-1	NSID-2	UC	NSID-1	NSID-2	UC
Chi-Chi	6.238 (23.871)	6.156 (24.872)	8.194	19.136 (32.750)	19.897 (30.076)	28.455
Imperial_Valley	12.934 (29.156)	13.760 (24.632)	18.257	24.944 (78.000)	54.362 (52.054)	113.382
Landers	24.773 (31.209)	26.623 (26.072)	36.012	23.103 (67.203)	37.809 (46.327)	70.443
Kocaeli	14.160 (49.903)	15.051 (46.750)	28.265	31.854 (65.560)	33.397 (63.892)	92.491
RGB1	24.707 (67.601)	21.268 (72.111)	76.259	21.301 (78.184)	33.937 (65.243)	97.64
RGB2	36.952 (63.694)	31.618 (68.935)	101.779	35.104 (80.860)	56.728 (69.070)	183.406

In this table, (1) the “#” in (#) indicates the percentage decrease in the peak displacement of the vibration control system based on NSID-1 and NSID-2 compared to the peak displacement of the uncontrolled system; (2) UC represents uncontrolled system; (3) # =(UC-NSID-1 or NSID-2)∗100%/UC.

**Table 9:** Root mean square displacement time histories of the top floors of the adjacent MDOF building structures under different seismic excitation.

Earthquake	Left building (mm)			Right building (mm)		
	NSID-1	NSID-2	UC	NSID-1	NSID-2	UC
Chi-Chi	1.063 (42.260)	0.892 (51.548)	1.841	1.537 (85.070)	3.322 (67.732)	10.295
Imperial_Valley	2.282 (47.016)	2.513 (41.653)	4.307	4.718 (88.605)	13.368 (67.714)	41.405
Landers	3.588 (66.691)	3.631 (66.292)	10.772	3.499 (83.748)	5.908 (72.559)	21.530
Kocaeli	2.728 (75.139)	2.666 (75.704)	10.973	5.383 (87.307)	6.503 (84.666)	42.408
RGB1	7.176 (78.543)	5.773 (82.738)	33.444	5.811 (81.555)	10.364 (67.104)	31.505
RGB2	10.109 (75.271)	9.249 (77.375)	40.879	9.899 (84.982)	15.504 (76.478)	65.914

In this table, (1) the “#” in (#) indicates the percentage decrease in the root mean square displacements of the vibration control system based on NSID-1 and NSID-2 compared to the root mean square displacement of the uncontrolled system; (2) UC represents uncontrolled system; (3) # =(UC-NSID-1 or NSID-2)∗100%/UC.

**Figure 28:**
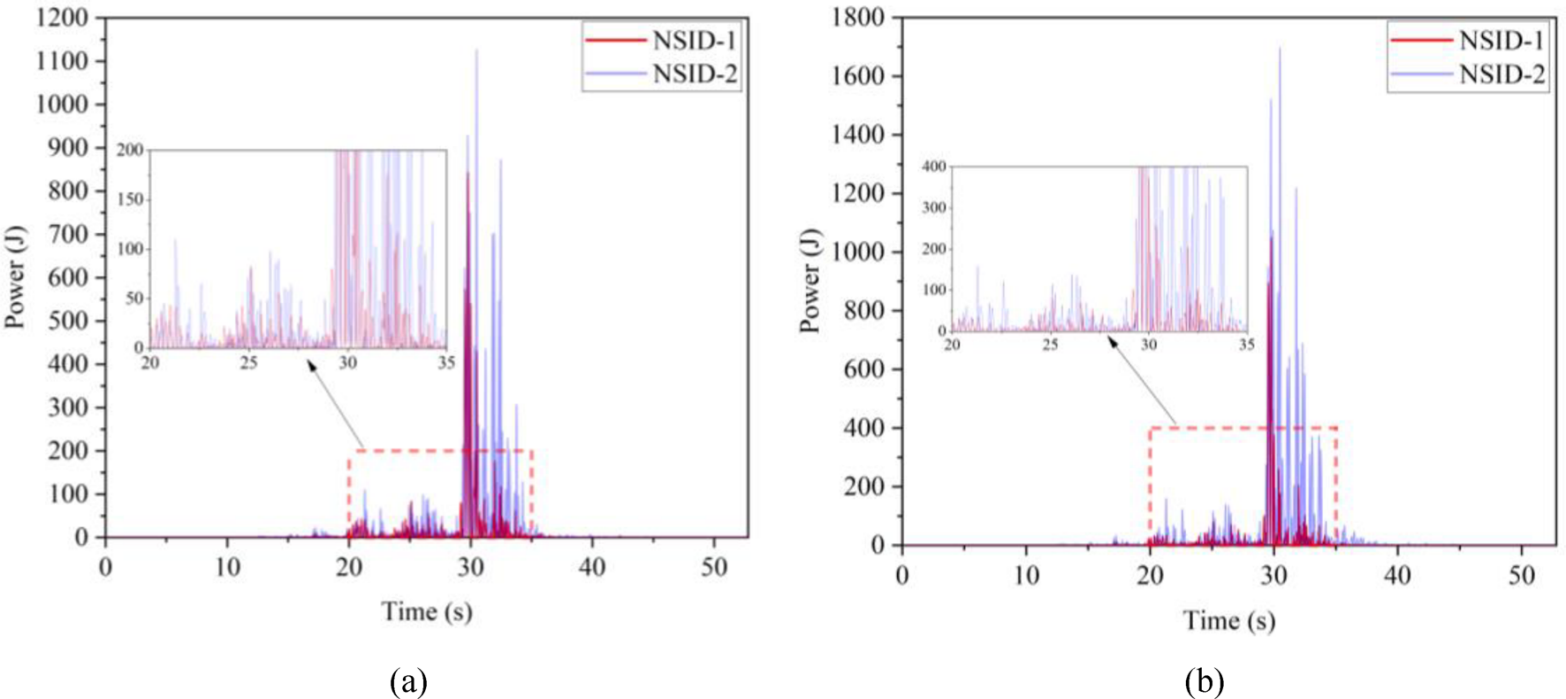
Kinetic energy of the control device in the ground floor under Chi-Chi seismic excitation. (**a**) Optimizing the left building structure. (**b**) Optimizing the right uilding structure

**Figure 29:**
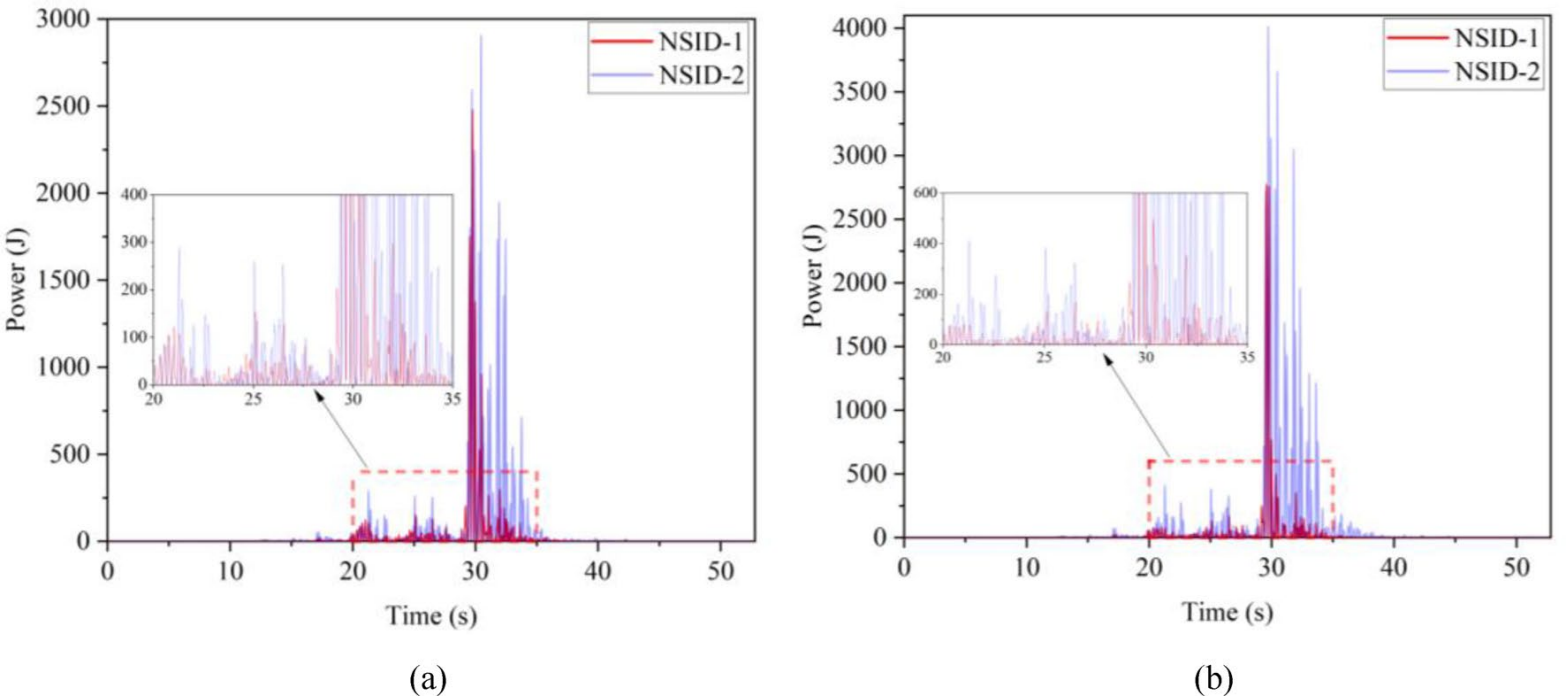
Kinetic energy of the control device in the second floor under Chi-Chi seismic excitation. (**a**) Optimizing the left building structure. (**b**) Optimizing the right uilding structure

**Figure 30:**
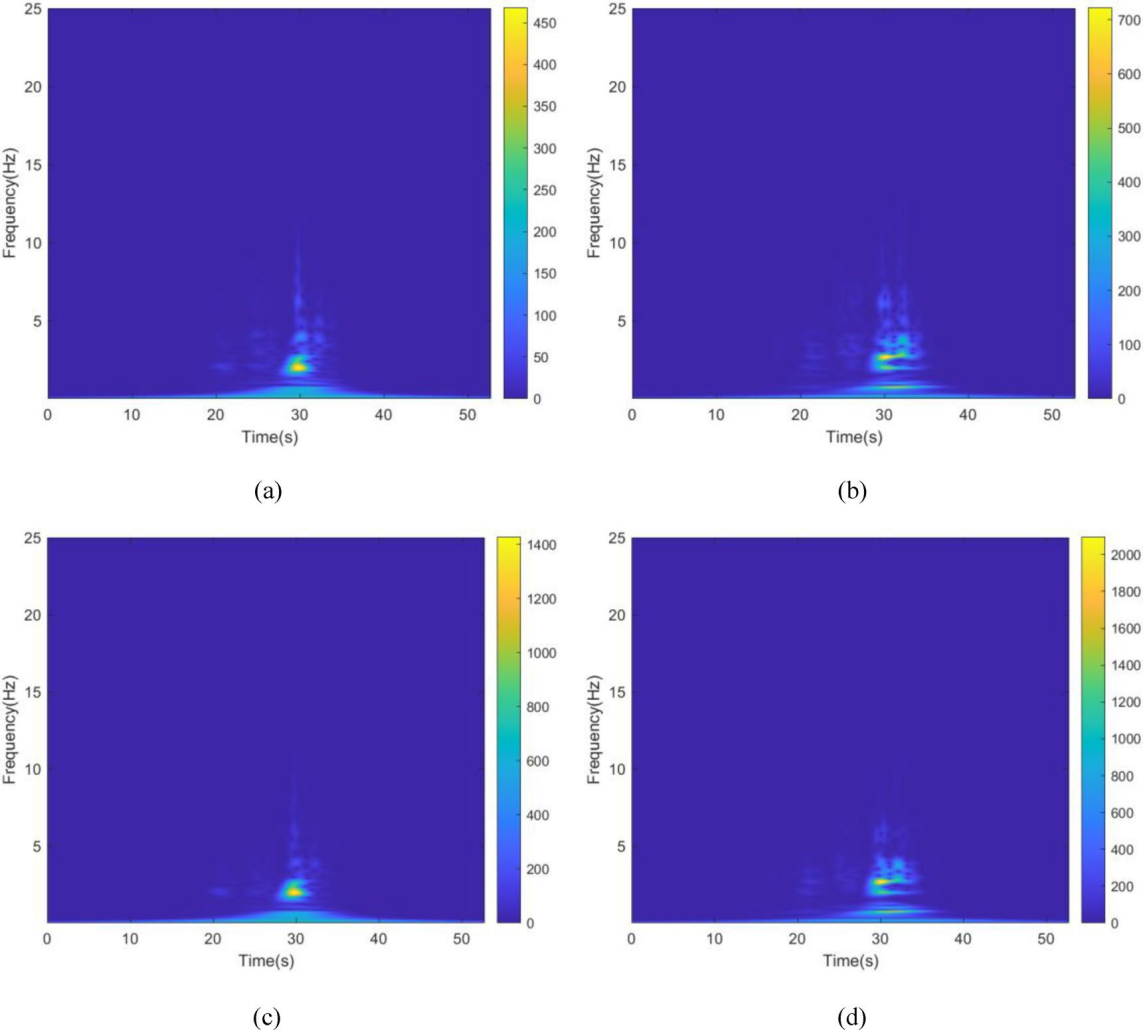
Time–frequency energy diagram of the inerter when optimizing the left building structure. (**a**) The ground floor of the system based on NSID-1. (**b**) The ground floor of the system based on NSID-2. (**c**) The second floor of the system based on NSID-1. (**d**) The second floor of the system based on NSID-2

**Figure 31:**
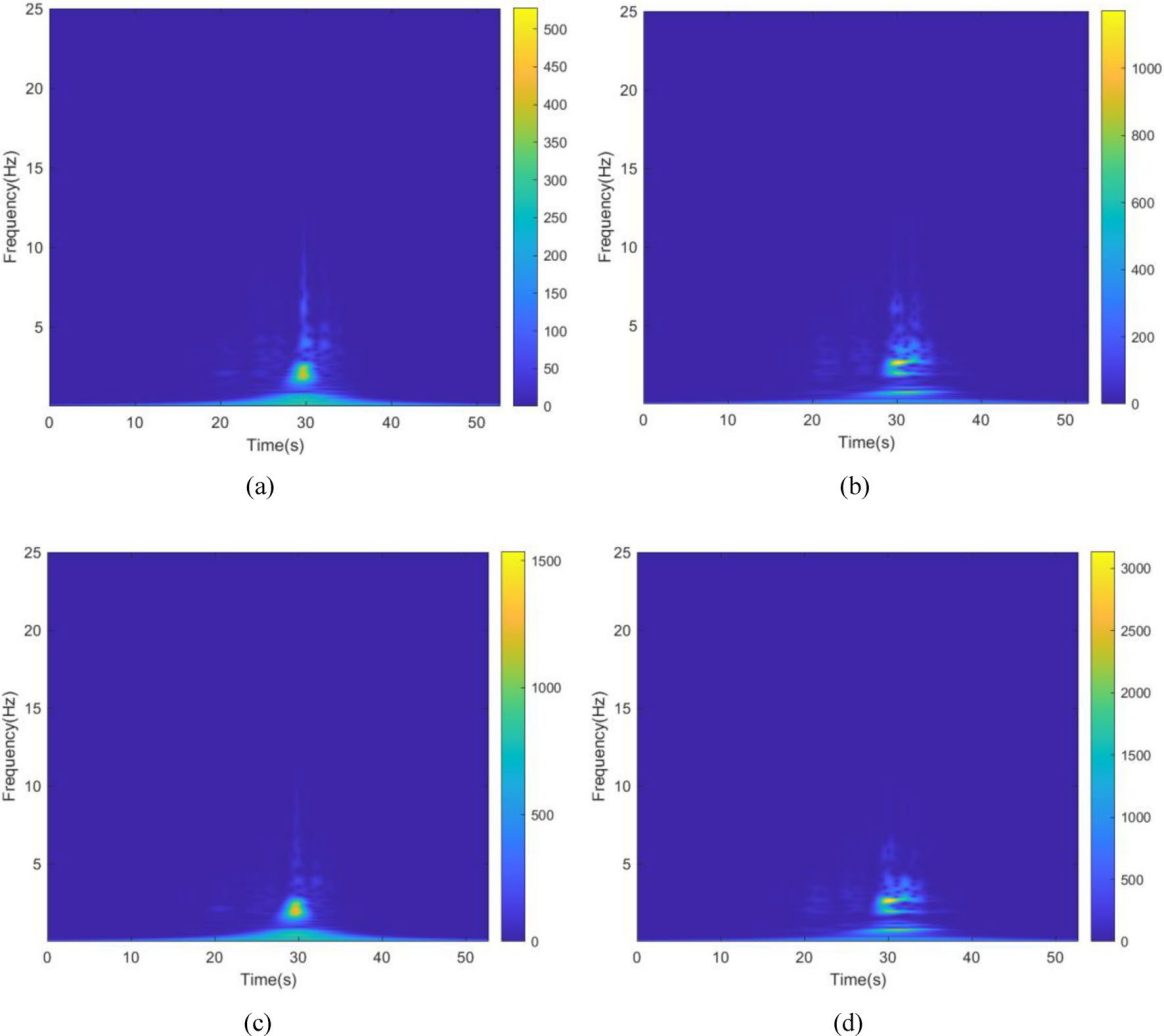
Time–frequency energy diagram of the inerter when optimizing the right building structure. (**a**) The ground floor of the system based on NSID-1. (**b**) The ground floor of the system based on NSID-2. (**c**) The second floor of the system based on NSID-1. (**d**) The second floor of the system based on NSID-2

The kinetic energy of the inerter in the vibration control system in each story under Chi-Chi seismic excitation is shown in Figs. 28 and 29. The peak and RMS of kinetic energy of inerter in each story under multiple seismic actions are shown in Tables 10 and 11. The kinetic energy of the inerters in the vibration control system based on NSID-2 is significantly larger compared to NSID-1. This means that the system with NSID-1 consumes energy mainly through dampers, while the system with NSID-2 converts seismic energy mainly into kinetic energy of inerter. Therefore, it would be more advantageous to use NSID-2 when considering energy harvesting in the vibration control system of adjacent MDOF structures. The time–frequency energy diagrams of the inerters in the two vibration control systems are shown in Figs. 30 and 31. The energy is mainly concentrated in the lower frequency region, while the peak energy is located near 2.5 Hz. In addition, the peak kinetic energy of the two MDOF structures with NSID-1 and NSID-2 corresponds to a lower frequency compared to the two SDOF structures.

**Table 10:** The peak and Root mean square (RMS) of Kinetic energy of the inerter in the ground floor.

Earthquake	Peak kinetic energy of inerter at optimizing the left building structure (J)		Peak kinetic energy of inerter at optimizing the right building structure (J)	
	NSID-1	NSID-2	NSID-1	NSID-2
Chi-Chi	843.334 (50.470)	1127.160 (83.200)	1050.591 (58.378)	1696.775 (121.400)
Imperial_Valley	2236.374 (219.699)	4973.666 (481.627)	1984.968 (217.063)	7039.592 (674.685)
Landers	4875.511 (257.514)	12,935.073 (750.672)	4617.702 (284.791)	13,382.764 (768.806)
Kocaeli	1148.509 (123.806)	2752.212 (255.982)	1722.799 (162.036)	3443.937 (297.609)
RGB1	1863.967 (239.604)	7805.490 (849.518)	2194.642 (306.648)	8137.625 (987.670)
RGB2	5533.628 (789.478)	12,060.128 (1855.298)	6883.294 (752.374)	16,924.734 (2565.562)

In this table, the “#” in (#) indicates the RMS of Kinetic energy of the inerter in the ground floor.

**Table 11:** The peak and Root mean square (RMS) of Kinetic energy of the inerter in the second floor.

Earthquake	Peak kinetic energy of inerter at optimizing the left building structure (J)		Peak kinetic energy of inerter at optimizing the right building structure (J)	
	NSID-1	NSID-2	NSID-1	NSID-2
Chi-Chi	2480.633 (146.642)	2905.589 (226.650)	2773.400269 (167.868)	4013.082487 (327.907)
Imperial_Valley	6977.605984 (645.246)	12,119.32769 (1313.524)	6155.219529 (591.479)	14,213.68191 (1814.142)
Landers	5849.459922 (405.354)	24,014.47584 (1650.945)	5322.33427 (472.985)	30,865.4881 (2070.012)
Kocaeli	3141.535086 (333.180)	7667.535952 (692.289)	4586.526342 (428.352)	9197.711764 (792.492)
RGB1	4468 (606.368)	17,054.65 (2102.449)	4493.0403 (682.175)	19,584.82 (2406.653)
RGB2	15,279.31 (2259.357)	31,334.16 (4763.722)	17,230.898 (1850.007)	42,805.05 (6473.246)

In this table, the “#” in (#) indicates the RMS of Kinetic energy of the inerter in the second floor.

## Conclusions

This paper discusses the seismic performance of vibration control systems based on NSID-1 and NSID-2. The influence of parameter variations on the dynamic characteristics of the two vibration control systems is analyzed and compared in order to make the systems have better robustness and stability. The design parameters (negative stiffness ratio $\boldsymbol{\alpha }$ and damping ratio ${{\varvec{\xi}}}_{{\varvec{b}}}$) of the vibration control system are optimized using the *H*_2_ norm theory and Monte Carlo pattern search method in order to achieve the best effect of vibration control for the system. Finally, the displacement time histories of adjacent building structures under several seismic excitations are compared. The following conclusions are obtained:

1. The frequency response function of a building structure increases significantly when the masses of adjacent building structures are close to each other. At the same time, when the building mass ratio is too large, it will also lead to an increase in the frequency response function of the building structure. Therefore, appropriately increasing the difference in the mass of adjacent building structures can effectively improve the vibration control performance of the system and reduce the vibration displacement caused by external excitation.
2. The ratio of adjacent building structures stiffnesses has a significant influence on the frequency ratio that corresponds to the peak value of the building frequency response function, which results in the building structures exhibiting significant differences in seismic performance against different types of seismic waves (short-period and long-period waves). It should be noted that the robustness and stability of the vibration control systems based on NSID-1 and NSID-2 are significantly degraded when the ratio of the frequencies of the adjacent building structures is close to 1. Therefore, the stiffness and mass of adjacent building structures need to be considered together to avoid the frequency ratio of adjacent building structures approaching 1.
3. As a control device connecting two building structures, its own mass can significantly influence the seismic performance of the vibration control system in the adjacent building. Although a small inerter mass ratio can effectively control the cost, too low inerter mass ratio will lead to a significant increase in the amplitude of the frequency response function of the vibration control systems.
4. Although both NSID-1 and NSID-2 are effective in improving the seismic performance of adjacent building structures, the two vibration control devices reduce seismic damage to building structures in different ways. Because NSID-2 can convert most of the seismic energy into kinetic energy, NSID-2 has a good potential for application in installations where both vibration control and energy harvesting are considered.

### Supplementary Information

Supplementary Information.

## Data Availability

The datasets generated and/or analyzed during the current study are not publicly available because all data are presented in the article and therefore, there is no need to include raw data but they are available from the corresponding author upon reasonable request.
