# Supplementary material for: Parameter optimization of vibration control system for adjacent building structures based on negative stiffness inerter damper
Source: Sci Rep. 2024 Apr 16;14:8742. doi: 10.1038/s41598-024-59380-1 (PMC11021558; doi:10.1038/s41598-024-59380-1)
Supplement: Supplementary file 1 — Supplementary Information. [file 41598_2024_59380_MOESM1_ESM.docx]

Appendix A

The numerator and denominator of the displacement frequency response function $H_{l}^{NSID-1}$ are ${b_{0}^{1}\sim b}_{5}^{1}$ and $a_{0}^{1}\sim a_{6}^{1}$, respectively.

$$a_{6}^{1}=1$$

$$a_{5}^{1}=2(f_{b}(1+\mu_{b}+\mu_{b}\mu_{l})\xi_{b}+\xi_{l}+f_{r}\xi_{r})$$

$$a_{4}^{1}=1+{f_{r}}^{2}(1+\beta)+{f_{b}}^{2}(1+\alpha+\mu_{b})+4f_{r}\xi_{l}\xi_{r}+4f_{b}\xi_{b}(\xi_{l}+\mu_{b}\mu_{l}\xi_{l}+f_{r}(1+\mu_{b})\xi_{r}))$$

$$a_{3}^{1}=2({f_{b}}^{3}\alpha\mu_{b}(1+\mu_{l})\xi_{b}+f_{r}(f_{r}(1+\beta)\xi_{l}+\xi_{r})+{f_{b}}^{2}((1+\alpha)\xi_{l}+f_{r}(1+\alpha+\mu_{b})\xi_{r})+f_{b}\xi_{b}(1+{f_{r}}^{2}(1+\mu_{b})+\mu_{b}\mu_{l}+4f_{r}\xi_{l}\xi_{r}))$$

$$a_{2}^{1}=({f_{r}}^{2}(1+\beta)+{f_{b}}^{4}\alpha\mu_{b}+4f_{b}f_{r}\xi_{b}(f_{r}\xi_{l}+\xi_{r})+4{f_{b}}^{3}\alpha\mu_{b}\xi_{b}(\mu_{l}\xi_{l}+f_{r}\xi_{r})+{f_{b}}^{2}(1+\alpha+{f_{r}}^{2}(1+\alpha+\alpha\beta+\mu_{b})+4f_{r}(1+\alpha)\xi_{l}\xi_{r}))$$

$$a_{1}^{1}=2f_{b}({f_{b}}^{2}\alpha\mu_{b}\mu_{l}\xi_{b}+{f_{r}}^{2}(\xi_{b}+{f_{b}}^{2}\alpha\mu_{b}\xi_{b}+f_{b}(1+\alpha+\alpha\beta)\xi_{l})+f_{b}f_{r}(1+\alpha+{f_{b}}^{2}\alpha\mu_{b})\xi_{r})$$

$$a_{0}^{1}={f_{b}}^{2}{f_{r}}^{2}(1+\alpha(1+\beta+{f_{b}}^{2}\mu_{b}))$$

$$b_{4}^{1}=-1$$

$$b_{3}^{1}=-2(f_{b}(1+\mu_{b}+\mu_{b}\mu_{l})\xi_{b}+f_{r}\xi_{r})$$

$$b_{2}^{1}=-{f_{r}}^{2}(1+\beta)-{f_{b}}^{2}(1+\alpha+\mu_{b})-4f_{b}f_{r}\xi_{b}\xi_{r}$$

$$b_{1}^{1}=-2f_{b}({f_{r}}^{2}\xi_{b}+{f_{b}}^{2}\alpha\mu_{b}(1+\mu_{l})\xi_{b}+f_{b}f_{r}(1+\alpha)\xi_{r})$$

$$b_{0}^{1}=-{f_{b}}^{2}{f_{r}}^{2}(1+\alpha+\alpha\beta)-{f_{b}}^{4}\alpha\mu_{b}$$

The numerator and denominator of the displacement frequency response function $H_{r}^{NSID-1}$ are $d_{0}^{1}\sim d_{5}^{1}$ and $c_{0}^{1}\sim c_{6}^{1}$, respectively.

$$c_{6}^{1}=-1$$

$$c_{5}^{1}=-2f_{b}(1+\mu_{b})\xi_{b}-2\xi_{l}+2\mu_{b}(f_{b}\mu_{b}\mu_{l}\xi_{b}+f_{r}\xi_{r})-2(1+\mu_{b})(f_{b}\mu_{b}\mu_{l}\xi_{b}+f_{r}\xi_{r})$$

$$c_{4}^{1}=-1+{f_{b}}^{2}\alpha\mu_{b}+{f_{r}}^{2}(1+\beta)\mu_{b}-{f_{b}}^{2}(1+\alpha)(1+\mu_{b})-{f_{r}}^{2}(1+\beta)(1+\mu_{b})+4{f_{b}}^{2}\mu_{b}(1+\mu_{b})\mu l{\xi_{b}}^{2}-4f_{b}\xi_{b}\xi_{l}-4f_{b}(1+\mu_{b})\xi_{b}(f_{b}\mu_{b}\mu_{l}\xi_{b}+f_{r}\xi_{r})-4\xi_{l}(f_{b}\mu_{b}\mu_{l}\xi_{b}+f_{r}\xi_{r})$$

$$c_{3}^{1}=-2f_{b}\xi_{b}-2{f_{b}}^{3}\alpha\mu_{b}\xi_{b}+2f_{b}{f_{r}}^{2}\beta(1+\mu_{b})\xi_{b}-2f_{b}{f_{r}}^{2}(1+\beta)(1+\mu_{b})\xi_{b}+2{f_{b}}^{3}\mu_{b}(1+\mu_{b})\mu_{l}\xi_{b}-2{f_{b}}^{2}(1+\alpha)\xi_{l}-2{f_{r}}^{2}(1+\beta)\xi_{l}+8{f_{b}}^{2}\mu_{b}\mu_{l}{\xi_{b}}^{2}\xi_{l}-2(f_{b}\mu_{b}\mu_{l}\xi_{b}+f_{r}\xi_{r})+2{f_{b}}^{2}\alpha\mu_{b}(f_{b}\mu_{b}\mu_{l}\xi_{b}+f_{r}\xi_{r})-2{f_{b}}^{2}(1+\alpha)(1+\mu_{b})(f_{b}\mu_{b}\mu_{l}\xi_{b}+f_{r}\xi_{r})-8f_{b}\xi_{b}\xi_{l}(f_{b}\mu_{b}\mu_{l}\xi_{b}+f_{r}\xi_{r})$$

$$c_{2}^{1}=-{f_{b}}^{2}(1+\alpha)-{f_{r}}^{2}(1+\beta)+{f_{b}}^{4}\alpha^{2}\mu_{b}-{f_{b}}^{4}\alpha(1+\alpha)\mu_{b}+{f_{b}}^{2}{f_{r}}^{2}\alpha(1+\beta)\mu_{b}+{f_{b}}^{2}{f_{r}}^{2}\beta(1+\mu_{b})-{f_{b}}^{2}{f_{r}}^{2}(1+\alpha)(1+\beta)(1+\mu_{b})+4{f_{b}}^{2}\mu_{b}\mu_{l}{\xi_{b}}^{2}+4{f_{b}}^{4}\alpha{\mu_{b}}^{2}\mu_{l}{\xi_{b}}^{2}+4f_{b}{f_{r}}^{2}\beta\xi_{b}\xi_{l}-4f_{b}{f_{r}}^{2}(1+\beta)\xi_{b}\xi_{l}+4{f_{b}}^{3}\mu_{b}\mu_{l}\xi_{b}\xi_{l}-4f_{b}\xi_{b}(f_{b}\mu_{b}\mu_{l}\xi_{b}+f_{r}\xi_{r})-4{f_{b}}^{3}\alpha\mu_{b}\xi_{b}(f_{b}\mu_{b}\mu_{l}\xi_{b}+f_{r}\xi_{r})-4{f_{b}}^{2}(1+\alpha)\xi_{l}(f_{b}\mu_{b}\mu_{l}\xi_{b}+f_{r}\xi_{r})$$

$$c_{1}^{1}=2f_{b}{f_{r}}^{2}\beta\xi_{b}-2f_{b}{f_{r}}^{2}(1+\beta)\xi_{b}+2{f_{b}}^{3}{f_{r}}^{2}\alpha\beta\mu_{b}\xi_{b}-2{f_{b}}^{3}{f_{r}}^{2}\alpha(1+\beta)\mu_{b}\xi_{b}+2{f_{b}}^{3}\mu_{b}\mu_{l}\xi_{b}+2{f_{b}}^{5}\alpha{\mu_{b}}^{2}\mu_{l}\xi_{b}+2{f_{b}}^{2}{f_{r}}^{2}\beta\xi_{l}-2{f_{b}}^{2}{f_{r}}^{2}(1+\alpha)(1+\beta)\xi_{l}-2{f_{b}}^{2}(1+\alpha)(f_{b}\mu_{b}\mu_{l}\xi_{b}+f_{r}\xi_{r})+2{f_{b}}^{4}\alpha^{2}\mu_{b}(f_{b}\mu_{b}\mu_{l}\xi_{b}+fr\xi_{r})-2{f_{b}}^{4}\alpha(1+\alpha)\mu_{b}(f_{b}\mu_{b}\mu_{l}\xi_{b}+f_{r}\xi_{r})$$

$$c_{0}^{1}={f_{b}}^{2}{f_{r}}^{2}\beta-{f_{b}}^{2}{f_{r}}^{2}(1+\alpha)(1+\beta)+{f_{b}}^{4}{f_{r}}^{2}\alpha\beta\mu_{b}+{f_{b}}^{4}{f_{r}}^{2}\alpha^{2}(1+\beta)\mu_{b}-{f_{b}}^{4}{f_{r}}^{2}\alpha(1+\alpha)(1+\beta)\mu_{b}$$

$$d_{4}^{1}=1$$

$$d_{3}^{1}=2(f_{b}(1+\mu_{b}+\mu_{b}\mu_{l})\xi_{b}+\xi_{l})$$

$$d_{2}^{1}=1+{f_{r}}^{2}\beta+{f_{b}}^{2}(1+\alpha+\mu_{b})+4f_{b}\xi_{b}\xi_{l}$$

$$d_{1}^{1}=2f_{b}(\xi_{b}+{f_{b}}^{2}\alpha\mu_{b}(1+\mu_{l})\xi_{b}+f_{b}(1+\alpha)\xi_{l})$$

$$d_{0}^{1}={f_{b}}^{2}(1+\alpha+{f_{r}}^{2}\alpha\beta+{f_{b}}^{2}\alpha\mu_{b})$$

Appendix B

Taking the calculation process of the H2 norm $\mathrm{PI}_{i}^{NSID-1}$ of the displacement frequency response function $H_{i}^{NSID-1}$ as an example:

$$\mathrm{PI}_{i}^{NSID-1}=\int_{-\infty}^{+\infty} \left| H_{i}^{NSID-1} \right|^{2}d\lambda=\frac{Q_{i}^{NSID-1}}{V_{i}^{NSID-1}}$$

Where

$$Q_{i}^{NSID-1}=-a_{2}^{1}a_{3}^{1}a_{4}^{1}a_{5}^{1}a_{6}^{1}v_{0}^{1}+a_{1}^{1}{a_{4}^{1}}^{2}a_{5}^{1}a_{6}^{1}v_{0}^{1}+{a_{2}^{1}}^{2}{a_{5}^{1}}^{2}a_{6}^{1}v_{0}^{1}-a_{0}^{1}a_{4}^{1}{a_{5}^{1}}^{2}a_{6}^{1}v_{0}^{1}+a_{2}^{1}{a_{3}^{1}}^{2}{a_{6}^{1}}^{2}v_{0}^{1}-a_{1}^{1}a_{3}^{1}a_{4}^{1}{a_{6}^{1}}^{2}v_{0}^{1}-2a_{1}^{1}a_{2}^{1}a_{5}^{1}{a_{6}^{1}}^{2}v_{0}^{1}+a_{0}^{1}a_{3}^{1}a_{5}^{1}{a_{6}^{1}}^{2}v_{0}^{1}+{a_{1}^{1}}^{2}{a_{6}^{1}}^{3}v_{0}^{1}+a_{0}^{1}a_{3}^{1}a_{4}^{1}a_{5}^{1}a_{6}^{1}v_{1}^{1}-a_{0}^{1}a_{2}^{1}{a_{5}^{1}}^{2}a_{6}^{1}v_{1}^{1}-a_{0}^{1}{a_{3}^{1}}^{2}{a_{6}^{1}}^{2}v_{1}^{1}+a_{0}^{1}a1a_{5}^{1}{a_{6}^{1}}^{2}v_{1}^{1}-a_{0}^{1}a_{1}^{1}a_{4}^{1}a_{5}^{1}a_{6}^{1}v_{2}^{1}+{a_{0}^{1}}^{2}{a_{5}^{1}}^{2}a_{6}^{1}v_{2}^{1}+a_{0}^{1}a_{1}^{1}a_{3}^{1}{a_{6}^{1}}^{2}v_{2}^{1}+a_{0}^{1}a_{1}^{1}a_{2}^{1}a_{5}^{1}a_{6}^{1}v_{3}^{1}-{a_{0}^{1}}^{2}a_{3}^{1}a_{5}^{1}a_{6}^{1}v_{3}^{1}-a_{0}^{1}{a_{1}^{1}}^{2}{a_{6}^{1}}^{2}v_{3}^{1}-a_{0}^{1}a_{1}^{1}a_{2}^{1}a_{3}^{1}a_{6}^{1}v_{4}^{1}+{a_{0}^{1}}^{2}{a_{3}^{1}}^{2}a_{6}^{1}v_{4}^{1}+a_{0}^{1}{a_{1}^{1}}^{2}a_{4}^{1}a_{6}^{1}v_{4}^{1}-{a_{0}^{1}}^{2}a_{1}^{1}a_{5}^{1}a_{6}^{1}v_{4}^{1}+a_{0}^{1}a_{1}^{1}a_{2}^{1}a_{3}^{1}a_{4}^{1}v_{5}^{1}-{a_{0}^{1}}^{2}{a_{3}^{1}}^{2}a_{4}^{1}v_{5}^{1}-a_{0}^{1}{a_{1}^{1}}^{2}{a_{4}^{1}}^{2}v_{5}^{1}-a_{0}^{1}a_{1}^{1}{a_{2}^{1}}^{2}a_{5}^{1}v_{5}^{1}+{a_{0}^{1}}^{2}a_{2}^{1}a_{3}^{1}a_{5}^{1}v_{5}^{1}+2{a_{0}^{1}}^{2}a_{1}^{1}a_{4}^{1}a_{5}^{1}v_{5}^{1}-{a_{0}^{1}}^{3}{a_{5}^{1}}^{2}v_{5}^{1}+a_{0}^{1}{a_{1}^{1}}^{2}a_{2}^{1}a_{6}^{1}v_{5}^{1}-{a_{0}^{1}}^{2}a_{1}^{1}a_{3}^{1}a_{6}^{1}v_{5}^{1}$$

$$V_{i}^{NSID-1}=2a_{6}^{1}(a_{0}^{1}a_{1}^{1}a_{2}^{1}a_{3}^{1}a_{4}^{1}a_{5}^{1}-{a_{0}^{1}}^{2}{a_{3}^{1}}^{2}a_{4}^{1}a_{5}^{1}-a_{0}^{1}{a_{1}^{1}}^{2}{a_{4}^{1}}^{2}a_{5}^{1}-a_{0}^{1}a_{1}^{1}{a_{2}^{1}}^{2}{a_{5}^{1}}^{2}+{a_{0}^{1}}^{2}a_{2}^{1}a_{3}^{1}{a_{5}^{1}}^{2}+2{a_{0}^{1}}^{2}a_{1}^{1}a_{4}^{1}{a_{5}^{1}}^{2}-{a_{0}^{1}}^{3}{a_{5}^{1}}^{3}-a_{0}^{1}a_{1}^{1}a_{2}^{1}{a_{3}^{1}}^{2}a_{6}^{1}+{a_{0}^{1}}^{2}{a_{3}^{1}}^{3}a_{6}^{1}+a_{0}^{1}{a_{1}^{1}}^{2}a_{3}^{1}a_{4}^{1}a_{6}^{1}+2a_{0}^{1}{a_{1}^{1}}^{2}a_{2}^{1}a_{5}^{1}a_{6}^{1}-3{a_{0}^{1}}^{2}a_{1}^{1}a_{3}^{1}a_{5}^{1}a_{6}^{1}-a_{0}^{1}{a_{1}^{1}}^{3}{a_{6}^{1}}^{2})$$

$$v_{0}^{1}={b_{0}^{1}}^{2}$$

$$v_{1}^{1}={b_{1}^{1}}^{2}-2b_{0}^{1}b_{2}^{1}$$

$$v_{2}^{1}={b_{2}^{1}}^{2}-2b_{1}^{1}b_{3}^{1}+2b_{0}^{1}b_{4}^{1}$$

$$v_{3}^{1}={b_{3}^{1}}^{2}-2b_{2}^{1}b_{4}^{1}$$

$$v_{4}^{1}={b_{4}^{1}}^{2}$$

$$v_{5}^{1}=0$$

Appendix C

The numerator and denominator of the displacement frequency response function $H_{l}^{NSID-2}$ are ${b_{0}^{2}\sim b}_{5}^{2}$ and $a_{0}^{2}\sim a_{6}^{2}$, respectively.

$$a_{6}^{2}=2\xi_{b}$$

$$a_{5}^{2}=f_{b}(1+\alpha)+4\xi_{b}(\xi_{l}+f_{r}\xi_{r})$$

$$a_{4}^{2}=2(f_{b}(1+\alpha)(\xi_{l}+f_{r}\xi_{r})+\xi_{b}(1+{fr}^{2}+{f_{b}}^{2}(1+\alpha+\mu_{b}+\mu_{b}\mu_{l})+4f_{r}\xi_{l}\xi_{r}))$$

$$a_{3}^{1}={f_{b}}^{3}\alpha\mu_{b}(1+\mu_{l})+4f_{r}\xi_{b}(f_{r}\xi_{l}+\xi_{r})+4{f_{b}}^{2}\xi_{b}((1+\alpha+\mu_{b}\mu_{l})\xi_{l}+f_{r}(1+\alpha+\mu_{b})\xi_{r})+f_{b}(1+\alpha)(1+{fr}^{2}+4f_{r}\xi_{l}\xi_{r})$$

$$a_{2}^{1}=2({f_{r}}^{2}\xi_{b}+{f_{b}}^{4}\alpha\mu_{b}(1+\mu_{l})\xi_{b}+f_{b}f_{r}(1+\alpha)(f_{r}\xi_{l}+\xi_{r})+{f_{b}}^{3}\alpha\mu_{b}(\mu_{l}\xi_{l}+f_{r}\xi_{r})+{f_{b}}^{2}\xi_{b}(1+\alpha+{f_{r}}^{2}(1+\alpha+\mu_{b})+\mu_{b}\mu_{l}+4f_{r}(1+\alpha)\xi_{l}\xi_{r}))$$

$$a_{1}^{1}=f_{b}({f_{b}}^{2}\alpha\mu_{b}\mu_{l}(1+4f_{b}\xi_{b}\xi_{l})+{f_{r}}^{2}(1+\alpha+{f_{b}}^{2}\alpha\mu_{b}+4f_{b}\xi_{b}\xi_{l}+4f_{b}\alpha\xi_{b}\xi_{l})+4f_{b}f_{r}(1+\alpha+{f_{b}}^{2}\alpha\mu_{b})\xi_{b}\xi_{r})$$

$$a_{0}^{1}=2{f_{b}}^{2}({f_{r}}^{2}(1+\alpha+{f_{b}}^{2}\alpha\mu_{b})+{f_{b}}^{2}\alpha\mu_{b}\mu_{l})\xi_{b}$$

$$b_{4}^{1}=-2\xi_{b}$$

$$b_{3}^{1}=-f_{b}-f_{b}\alpha-4f_{r}\xi_{b}\xi_{r}$$

$$b_{2}^{1}=-2({f_{r}}^{2}\xi_{b}+{f_{b}}^{2}(1+\alpha+\mu_{b}+\mu_{b}\mu_{l})\xi_{b}+f_{b}f_{r}(1+\alpha)\xi_{r})$$

$$b_{1}^{1}=-f_{b}({f_{r}}^{2}(1+\alpha)+{f_{b}}^{2}\alpha\mu_{b}(1+\mu_{l})+4f_{b}f_{r}(1+\alpha)\xi_{b}\xi_{r})$$

$$b_{0}^{1}=-2{f_{b}}^{2}({f_{r}}^{2}(1+\alpha)+{f_{b}}^{2}\alpha\mu_{b}(1+\mu_{l}))\xi_{b}$$

The numerator and denominator of the displacement frequency response function $H_{r}^{NSID-2}$ are $d_{0}^{2}\sim d_{5}^{2}$ and $c_{0}^{2}\sim c_{6}^{2}$, respectively.

$$c_{6}^{1}=2\xi_{b}$$

$$c_{5}^{1}=f_{b}(1+\alpha)+4\xi_{b}(\xi_{l}+f_{r}\xi_{r})$$

$$c_{4}^{1}=2(f_{b}(1+\alpha)(\xi_{l}+f_{r}\xi_{r})+\xi_{b}(1+{f_{r}}^{2}+{f_{b}}^{2}(1+\alpha+\mu_{b}+\mu_{b}\mu_{l})+4f_{r}\xi_{l}\xi_{r}))$$

$$c_{3}^{1}={f_{b}}^{3}\alpha\mu_{b}(1+\mu_{l})+4f_{r}\xi_{b}(f_{r}\xi_{l}+\xi_{r})+4{f_{b}}^{2}\xi_{b}((1+\alpha+\mu_{b}\mu_{l})\xi_{l}+f_{r}(1+\alpha+\mu_{b})\xi_{r})+f_{b}(1+\alpha)(1+{f_{r}}^{2}+4f_{r}\xi_{l}\xi_{r})$$

$$c_{2}^{1}=2({f_{r}}^{2}\xi_{b}+{f_{b}}^{4}\alpha\mu_{b}(1+\mu_{l})\xi_{b}+f_{b}f_{r}(1+\alpha)(f_{r}\xi_{l}+\xi_{r})+{f_{b}}^{3}\alpha\mu_{b}(\mu_{l}\xi_{l}+f_{r}\xi_{r})+{f_{b}}^{2}\xi_{b}(1+\alpha+{f_{r}}^{2}(1+\alpha+\mu_{b})+\mu_{b}\mu_{l}+4f_{r}(1+\alpha)\xi_{l}\xi_{r}))$$

$$c_{1}^{1}=f_{b}({f_{b}}^{2}\alpha\mu_{b}\mu_{l}(1+4f_{b}\xi_{b}\xi_{l})+{f_{r}}^{2}(1+\alpha+{f_{b}}^{2}\alpha\mu_{b}+4f_{b}\xi_{b}\xi_{l}+4f_{b}\alpha\xi_{b}\xi_{l})+4f_{b}f_{r}(1+\alpha+{f_{b}}^{2}\alpha\mu_{b})\xi_{b}\xi_{r})$$

$$c_{0}^{1}=2{f_{b}}^{2}({f_{r}}^{2}(1+\alpha+{f_{b}}^{2}\alpha\mu_{b})+{f_{b}}^{2}\alpha\mu_{b}\mu_{l})\xi_{b}$$

$$d_{4}^{1}=-2\xi_{b}$$

$$d_{3}^{1}=-f_{b}-f_{b}\alpha-4\xi_{b}\xi_{l}$$

$$d_{2}^{1}=-2(\xi_{b}+{f_{b}}^{2}(1+\alpha+\mu_{b}+\mu_{b}\mu_{l})\xi_{b}+f_{b}(1+\alpha)\xi_{l})$$

$$d_{1}^{1}=-f_{b}(1+\alpha+{f_{b}}^{2}\alpha\mu_{b}(1+\mu_{l})+4f_{b}\xi_{b}\xi_{l}+4f_{b}\alpha\xi_{b}\xi_{l})$$

$$d_{0}^{1}=-2{f_{b}}^{2}(1+\alpha+{f_{b}}^{2}\alpha\mu_{b}(1+\mu_{l}))\xi_{b}$$
